# *Papaver S*-determinants trigger mitochondrially derived ROS production and disrupt energy metabolism in incompatible pollen tubes

**DOI:** 10.1093/plcell/koag031

**Published:** 2026-02-19

**Authors:** Ludi Wang, An-Shan Hsiao, José Carli, Ali Raza, Zongcheng Lin, Dominique Arnaud, Julia M Davies, Vernonica E Franklin-Tong, Nicholas Smirnoff, Maurice Bosch

**Affiliations:** Institute of Biological, Environmental and Rural Sciences (IBERS), Aberystwyth University, Plas Gogerddan, Aberystwyth SY23 3EE, United Kingdom; Biosciences, Faculty of Health and Life Sciences, University of Exeter, Exeter EX4 4QD, United Kingdom; Institute of Biological, Environmental and Rural Sciences (IBERS), Aberystwyth University, Plas Gogerddan, Aberystwyth SY23 3EE, United Kingdom; Institute of Biological, Environmental and Rural Sciences (IBERS), Aberystwyth University, Plas Gogerddan, Aberystwyth SY23 3EE, United Kingdom; National Key Laboratory for Germplasm Innovation & Utilization of Horticultural Crops, College of Horticulture and Forestry Sciences, Huazhong Agricultural University, Wuhan 430070, China; Hubei Hongshan Laboratory, Wuhan 430070, China; Biosciences, Faculty of Health and Life Sciences, University of Exeter, Exeter EX4 4QD, United Kingdom; Department of Plant Sciences, University of Cambridge, Downing Street, Cambridge CB2 3EA, United Kingdom; School of Biosciences, College of Life and Environmental Sciences, University of Birmingham, Edgbaston, Birmingham B15 2TT, United Kingdom; Biosciences, Faculty of Health and Life Sciences, University of Exeter, Exeter EX4 4QD, United Kingdom; Institute of Biological, Environmental and Rural Sciences (IBERS), Aberystwyth University, Plas Gogerddan, Aberystwyth SY23 3EE, United Kingdom

## Abstract

Many plants use self-incompatibility (SI) mechanisms to prevent inbreeding. SI in *Papaver rhoeas* is triggered by allele-specific interaction between the pollen and pistil *S*-determinants, activating a Ca^2+^-dependent signaling network that leads to rapid reactive oxygen species (ROS) production and eventual programed cell death (PCD) in incompatible pollen. Expression of the *Papaver* pollen *S*-determinant (PrpS) in *Arabidopsis thaliana* recapitulates *Papaver* SI when challenged with the cognate pistil ligand (PrsS). Using roGFP2-Orp1, a genetically encoded hydrogen peroxide (H_2_O_2_) sensor, and measurements of mitochondrial metabolism, reveals a complex SI response. Within minutes, elevated cytosolic Ca^2+^ ([Ca^2+^]_cyt_) and cytosolic acidification converge to trigger mitochondrial H_2_O_2_ production, mitochondrial membrane depolarization, decreased respiration rate, and ATP depletion. In parallel, oxidative inactivation of GAPDH inhibits glycolysis, resulting in decreased TCA cycle intermediates and providing a feedback loop to enhance mitochondrial disruption. Preceding mitochondrial ROS production, SI rapidly arrests pollen tube growth *via* inactivation of plasma membrane-localized NADPH oxidase (RBOH) mediated superoxide production. This provides insights into how ROS signatures from NADPH oxidase and mitochondria drive distinct processes. We demonstrate that early mitochondrial disruption, likely driven by interconnected Ca^2+^, pH, and redox signaling, is a central feature of this SI response, underpinning rapid disruption of energy metabolism in incompatible pollen tubes prior to PCD.

## Teaser

Self-incompatibility triggers rapid disruption of mitochondrial function, resulting in mitochondrial H_2_O_2_ production, decreased respiration rate, and ATP depletion in incompatible pollen tubes.

## Introduction

Self-incompatibility (SI) is a pollen-pistil recognition system utilized by many flowering plants to prevent self-fertilization and maintain genetic diversity. This system is governed by tightly linked, polymorphic *S*-determinants expressed in the pollen and the pistil, which regulate mating compatibility and prevent inbreeding. In *Papaver rhoeas* (poppy), the *S*-determinants are the stigma-expressed S-protein (PrsS), which is secreted and acts as the ligand, and its pollen-specific transmembrane receptor (PrpS) (Foote et al. 1994; Wheeler et al. 2009). Interaction between these two proteins triggers a rapid growth arrest of incompatible pollen tubes mediated by a cytosolic free calcium ([Ca^2+^]_cyt_)-dependent signaling network that includes increases in reactive oxygen species (ROS), cytosolic acidification, and rapid ATP depletion (Wilkins et al. 2011, 2015; Wang et al. 2022; Bosch and Franklin-Tong 2024). These events trigger programed cell death (PCD) of incompatible pollen tubes several hours later (Thomas and Franklin-Tong 2004; Wang et al. 2019). Understanding how these molecular and cellular events coordinate pollen compatibility and control cell survival or death provides key insights into plant reproductive regulation and offers broader implications for manipulating crop fertility and breeding strategies.

SI in *Papaver* stimulates increases in ROS levels throughout the entire pollen tube, as demonstrated by the non-specific ROS-sensitive probe chloromethyl-2′7′-dichlorodihydrofluorescein diacetate acetyl ester (CM-H_2_DCFDA) (Hempel et al. 1999 ; Wilkins et al. 2011). This distinctive pattern of ROS accumulation raises important questions: What are the sources and targets of ROS during SI? To investigate these questions, a means of measuring ROS with greater spatial and temporal resolution is required. Recent advances in fluorescent biosensors, such as the H_2_O_2_-specific probe roGFP2-Orp1, now allow for precise and dynamic measurements of H_2_O_2_ levels (Nietzel et al. 2019). This sensor has been effectively used to study H_2_O_2_ production during pattern-triggered immunity (PTI) responses (Arnaud et al. 2023) and mitochondrial retrograde signaling (Khan et al. 2024) in *Arabidopsis*. Notably, the *Papaver* SI system has been functionally transferred to *Arabidopsis thaliana* (de Graaf et al. 2012; Lin et al. 2015; Wang et al. 2020, 2022). This model system, utilizing pollen tubes expressing PrpS, combined with the genetically encoded fluorescent probe roGFP2-Orp1, offers a powerful platform to investigate SI-induced subcellular H_2_O_2_ dynamics in greater depth using live-cell imaging approaches.

Pollen tube growth is an energy-intensive process, requiring high ATP levels to fuel the cellular machinery essential for tip growth (Rounds et al. 2011). We recently established that SI triggers a rapid and reduction in ATP levels (Wang et al. 2022) and that SI-induced ROS triggers irreversible oxidation of multiple enzymes, including those involved in energy metabolism (Haque et al. 2020). Reduced ATP synthesis implicates respiratory metabolism and mitochondria as early targets in the SI-induced PCD pathway. Supporting this, our previous studies revealed that SI triggers a rapid release of cytochrome *c* (Thomas and Franklin-Tong 2004) and leads to significant alterations in mitochondrial morphology (Geitmann et al. 2004) in *Papaver* pollen tubes.

Cytosolic pH ([pH]_cyt_) and [Ca^2+^]_cyt_ also play crucial roles in pollen tube growth. Under normal conditions, pollen tubes exhibit an apical gradient of both [pH]_cyt_ and [Ca^2+^]_cyt_, which mirror the oscillatory growth pattern characteristic of tip-growing cells (Holdaway-Clarke and Hepler 2003; Hepler et al. 2006). However, during SI, these tip-based ion gradients collapse, accompanied by elevated [Ca^2+^]_cyt_ (Franklin-Tong et al. 1993) and cytosolic acidification (Bosch and Franklin-Tong 2007; Wilkins et al. 2015; Wang et al. 2022), which causes cessation of growth and activation of PCD.

ROS, primarily superoxide generated *via* electron transport and oxidase enzymes, and its dismutation product hydrogen peroxide (H_2_O_2_), play a crucial role in plant development and stress responses (Smirnoff and Arnaud 2019), yet excessive ROS accumulation leads to PCD (Mittler et al. 2022). In plant cells that utilize polar growth, such as pollen tubes and root hairs, tip-localized superoxide production mediated by plasma membrane NADPH oxidases (RBOHs) is critical for sustained tip growth (Foreman et al. 2003; Potocký et al. 2007; Kaya et al. 2014; Smirnoff and Arnaud 2019). Recent evidence suggests that the intracellular origin of ROS plays a pivotal role in determining their signaling functions (Noctor and Foyer 2016; Mittler et al. 2022; Arnaud et al. 2023; Khan et al. 2024), adding further complexity to their regulation and underscoring the need for precise tools to dissect their roles.

In this study, we show that SI rapidly inhibits tip-localized superoxide production, concomitant with the inhibition of tip growth. Using the H_2_O_2_-specific sensor roGFP2-Orp1, we demonstrate increases in probe oxidation in the cytosol, mitochondria, and plastids. This reveals that ROS signals regulating growth and SI arise from distinct subcellular origins and exhibit unique spatial-temporal signatures. We also show that increases in [Ca^2+^]_cyt_ and cytosolic acidification contribute to SI-induced mitochondrial dysfunction and H_2_O_2_ generation. SI triggers a collapse in mitochondrial membrane potential, leading to reduced respiration and decreased levels of TCA cycle and glycolysis intermediates. Together, our findings provide critical insights into the interplay between [Ca^2+^]_cyt_, cytosolic pH, H_2_O_2,_ and mitochondria during SI, illustrating how these signals interact and converge to disrupt energy metabolism in pollen tubes.

## Results

### SI induces rapid inactivation of pollen tube tip-localized ROS production

**Figure 1:**
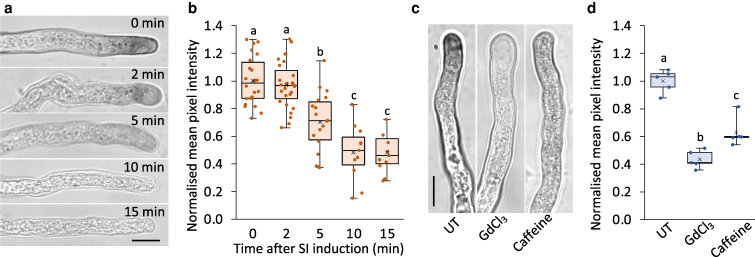
Inhibition of pollen tube tip-localized NADPH oxidase activity following SI induction. **a)** Detection of superoxide production in Arabidopsis pollen tubes during the SI response using nitroblue tetrazolium (NBT) staining. Representative pollen tubes expressing PrpS_1_ were stained with 5 mg ml^−1^ NBT at 0, 2, 5, 10, and 15 min after SI induction. Scale bar = 10 μm. **b)** Quantification of NBT staining intensity at pollen tube tips following SI induction. Reduced NBT staining corresponds to a decrease in pixel intensity. The mean pixel intensity at 0 min was normalized to 1 (n ≥ 10). NBT staining significantly decreased from 5 min after SI induction. **c)** Reduced tip-localized superoxide production detected by NBT staining after 2 min of treatment with 500 μM GdCl_3_ or 10 mM caffeine. Scale bar = 10 μm. **d)** Quantification of NBT staining intensity at pollen tube tips following treatment, with decreased pixel intensity indicating reduced superoxide production (n = 5). Different letters in **(b)** and **(d)** indicate significant (*P* < 0.05) differences based on Tukey's test. The box area of the boxplots contains the central 50% of values (first quartile to third quartile), whiskers indicate 1.5 times the interquartile range. The median is represented by a horizontal line, and the mean is indicated by a cross. Circles represent individual data points.

In our experimental system, SI was induced by applying recombinant PrsS_1_ protein to *in vitro* growing Arabidopsis pollen tubes expressing the *Papaver PrpS_1_* gene (de Graaf et al. 2012). Because SI causes increased ROS production in the pollen tube during SI (Wilkins et al. 2011), we measured production of tip-localized superoxide. Tip growth is dependent on ROS generated by NADPH oxidase activity (Potocký et al. 2007; Boisson-Dernier et al. 2013; Lassig et al. 2014; Kaya et al. 2015). Apoplastic superoxide production, driven by NADPH oxidases at the tip of growing pollen tubes, can be visualized by the reduction of nitroblue tetrazolium (NBT) to an insoluble blue formazan. NBT reduction is largely apoplastic and is abolished by knockdown of NADPH oxidase expression (Potocký et al. 2007). In untreated Arabidopsis pollen tubes, tip-localized formazan staining was observed (Fig. 1a), indicating superoxide production at the tip. Following SI-induction, tip-localized superoxide production was rapidly disrupted (Fig. 1a and b, Fig. S1). Within 5 min, the intensity of formazan staining was significantly reduced, and this reduction plateaued by 10 to 15 min (Fig. 1a and b). A similar response was observed in *Papaver* pollen tubes, where the basal level of apoplastic superoxide was higher, leading to a more pronounced decrease after SI induction (Fig. S1). In Arabidopsis, inactivation of NADPH oxidase corresponds to the rapid inhibition of tip growth during SI (Videos 1 and 2). To establish whether this disruption of NADPH oxidase activity was a consequence or cause of SI-induced growth inhibition, we treated Arabidopsis pollen tubes with 500 μM GdCl_3_ or 10 mM caffeine, which are known inhibitors of tip growth (Pierson et al. 1996; Geitmann et al. 2000). Within 2 min of treatment, we observed significant decreases in NBT staining (Fig. 1c and d), confirming that inhibition of tip growth rapidly inhibits tip-localized NADPH oxidase activity. As NADPH oxidase activity is inhibited by SI, abolishing tip-localized ROS production, it is evident that superoxide produced by NADPH oxidase cannot be the source of the SI-induced H_2_O_2_ in incompatible pollen tubes.

### Identifying the SI-induced intracellular ROS sources in pollen tubes

To investigate whether intracellular sources of ROS are involved in SI, we examined ROS production in subcellular compartments during SI using transgenic Arabidopsis lines co-expressing PrpS_1_ and roGFP2-Orp1. An Orp1 cysteine residue is oxidized by H_2_O_2,_ followed by a redox relay resulting in roGFP2 oxidation and a change in its fluorescence excitation spectrum, enabling ratiometric detection of its oxidation state (Nietzel et al. 2019). Because the probe is ratiometric, H_2_O_2_ measurements are independent of probe concentration across the pollen tubes. The roGFP2-Orp1 sensor, targeted to the cytosol, mitochondria, plastids, peroxisomes, and nuclei, allowed compartment-specific H_2_O_2_ detection. Fluorescence imaging confirmed the subcellular localization of the roGFP2-Orp1 sensor in growing pollen tubes (Fig. S2a). The oxidation of roGFP2-Orp1 by H_2_O_2_, measured ratiometrically as R_405/488_ (Nietzel et al. 2019), provides a quantitative readout of H_2_O_2_ dynamics. In untreated (UT) pollen tubes, roGFP2-Orp1 oxidation was relatively higher in mitochondria and peroxisomes, while the sensor was more reduced in the nuclei compared to the cytosol and plastids (Fig. S2b). The dynamic range (δ) of oxidation and reduction of roGFP2-Orp1 for each subcellular localization was assessed by treating pollen tubes with 15 mM H_2_O_2_ or 10 mM dithiothreitol (DTT; Fig. S2c-g). The response of roGFP2-Orp1 to pollen tubes treated with H_2_O_2_ was more pronounced in the cytosol and mitochondria, while DTT treatment resulted in a greater reduction in plastids and peroxisomes compared to cytosol, mitochondria, and nuclei. Despite nuclei (δ = 2.71) and peroxisomes (δ = 2.49) displaying a narrower dynamic range of roGFP2-Orp1 compared to the cytosol (δ = 3.65), mitochondria (δ = 5.09) and plastids (δ = 5.70), the probe characteristics are similar to previous reports and suitable for detecting changes in oxidation state (Nietzel et al. 2019; Ugalde et al. 2021; Arnaud et al. 2023).

**Figure 2:**
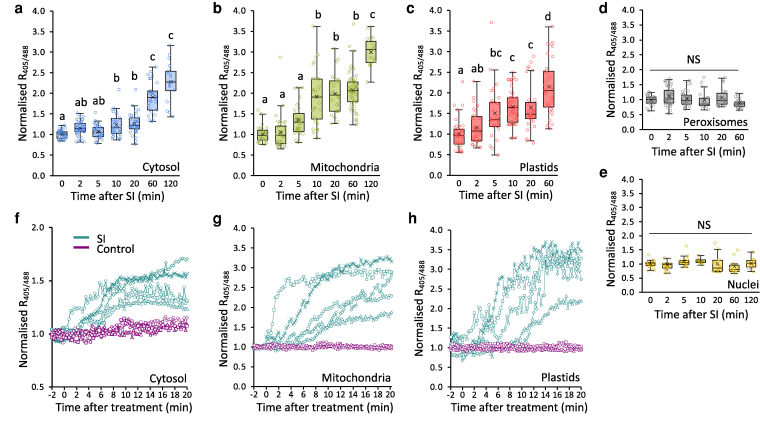
Changes in oxidation state of roGFP2-Orp1 targeted to various subcellular compartments in Arabidopsis pollen tubes. **a-e)** Quantification of R_405/488_ of roGFP2-Orp1 targeted to the cytosol **(a)**, mitochondria **(b)**, plastids **(c)**, peroxisomes **(d)**, and nuclei **(e)** after SI induction. **a-c)** R_405/488_ increased significantly (*P* < 0.001, one-way ANOVA) in the cytosol **(a)**, mitochondria **(b)**, and plastids **(c)** after SI induction compared with untreated (0 min) samples. **d, e)** No significant increase (NS, *P* > 0.05, one-way ANOVA) in R_405/488_ was detected in peroxisomes **(d)** or nuclei **(e)** after SI induction compared with untreated (0 min) samples. **f-h)** Quantification of R_405/488_ of roGFP2-Orp1 targeted to the cytosol **(f)**, mitochondria **(g)**, and plastids **(h)** during 0 to 20 min after SI induction (SI; n = 5) or treatment with growth medium (control; n = 3) in representative Arabidopsis pollen tubes expressing PrpS_1_ and roGFP2-Orp1. All R_405/488_ measurements were normalized to the mean ratio of untreated samples. For a-e, roGFP2-Orp1 was stabilized using 20 mM N-ethylmaleimide (NEM) at the end of each reaction. Different letters indicate significant differences based on Tukey's test. Sample sizes: **a)**: n = 16 to 42; **b)** n = 15 to 51; **c)** n = 29 to 49; **d)** n = 19 to 43; **e)** n = 8 to 21. The box area of the boxplots contains the central 50% of values (first quartile to third quartile), whiskers indicate 1.5 times the interquartile range. The median is represented by a horizontal line, and the mean is indicated by a cross. Circles represent individual data points.

**Figure 3:**
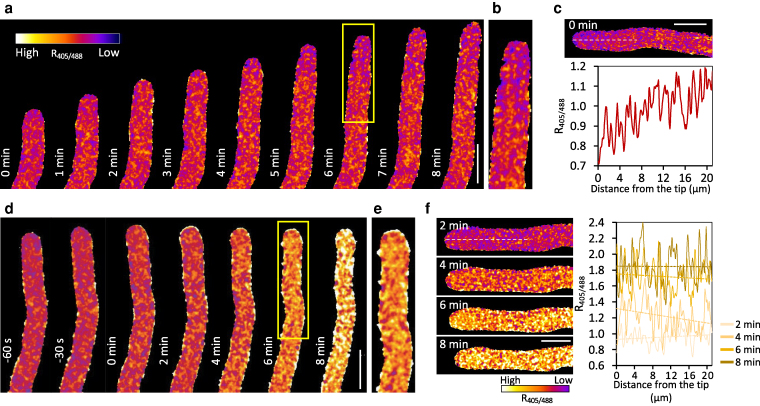
Increases in cytosolic roGFP2-Orp1 oxidation state in Arabidopsis pollen tubes during the SI response. **a, d)** Representative ratio images of Arabidopsis pollen tubes expressing PrpS_1_ and roGFP2-Orp1, either untreated **(a,** UT) or treated with PrsS_1_ to induce SI **(d,** SI). Scale bar = 10 μm. **b, e)** Enlarged views of the regions indicated by yellow boxes in **(a)** and **(d)**, showing the spatial variation in roGFP2-Orp1 oxidation before **(b)** and after SI induction **(e)**. Images in a and d were digitally extracted and made into a composite for comparison. **c, f)** Fluorescence ratio profiles of cytosolic roGFP2-Orp1 (R_405/488_) along the white dashed lines in a representative pollen tube without treatment **(c)** and after the SI induction **(f)**. The profiles illustrate SI induced dynamic changes in roGFP2-Orp1 oxidation state over time. Scale bar = 10 μm.

To examine the changes in oxidation state across various subcellular compartments following SI induction, we performed ratio-imaging of roGFP2-Orp1 by sampling populations of pollen tubes (Fig. 2). Probe oxidation is detected as an increase in the fluorescence excitation ratio (R_405/488_). We observed rapid increases in cytosolic and mitochondrial roGFP2-Orp1 oxidation, demonstrating an elevation in H_2_O_2_. In the cytosol, a significant increase in roGFP2-Orp1 oxidation was detectable as early as 10 min after SI induction compared to untreated pollen (Fig. 2a). At 60 min, increases in roGFP2-Orp1 oxidation became more pronounced, increasing further to 2.3-fold by 2 h after the SI induction (Fig. 2a). In mitochondria, a significant increase in probe oxidation was also observed as early as 10 min after SI induction reaching 3.0-fold at 120 min after SI induction (Fig. 2b). In plastids, significant increases in roGFP2-Orp1 oxidation state were detected from 5 min after SI induction onwards increasing by 2.2-fold 1 h after the SI induction (Fig. 2c). This demonstrates that SI stimulates increased H_2_O_2_ oxidation in the cytosol, mitochondria and plastids. SI did not stimulate roGFP2-Orp1 oxidation in all organelles; no significant alterations in R_405/488_ were measured in peroxisomes or nuclei (Fig. 2d and e). To obtain further information on early increases in roGFP2-Orp1 in the cytosol, mitochondria, and plastids following the SI induction, we tracked multiple individual pollen tubes over a 20-min period. While control pollen tubes treated with growth medium maintained a steady R_405/488_, the kinetics of R_405/488_ increases in SI-induced pollen tubes varied, but all increased within 10 min after induction (Fig. 2f and h). In untreated growing pollen tubes, representative time-lapse images showed that in the cytosol, roGFP2-Orp1 was more reduced in the tip region than in the shank, and no major changes in oxidation state were observed during growth (Fig. 3a-c, Video 1). After SI induction, this tip-to-shank difference rapidly disappeared, with a relatively uniform increase in roGFP2-Orp1 oxidation observed throughout the pollen tube (Fig. 3d-f, Video 2). This oxidation occurred shortly after the inhibition of tip growth (Fig. 3d, Video 2). This coincided with a redistribution of mitochondria: in growing pollen tubes, the tip “clear zone” is devoid of mitochondria, but following SI, the clear zone is lost, and mitochondria relocate into the tip region (Fig. S3a). The spatial correlation between mitochondrial redistribution and cytosolic sensor oxidation suggests that mitochondria may contribute as a source of H_2_O_2_. Consistent with this, SI induction also triggered a general increase in roGFP2-Orp1 oxidation in mitochondria and plastids, although no distinct spatial features were observed in these organelles in either growing or SI-induced pollen tubes (Fig. S3b and c).

To examine whether the SI-induced oxidation of roGFP2-Orp1 was solely due to increased H_2_O_2_ production or also involved impaired reduction capacity of the sensor due to other SI-induced events, we measured roGFP2-Orp1 oxidation following treatment with 2.5 mM H_2_O_2_, with or without a preceding SI induction. No significant differences in roGFP2-Orp1 oxidation were observed in the cytosol or mitochondria after H_2_O_2_ treatment, regardless of SI induction (Fig. S4a and b). However, in the plastids, roGFP2-Orp1 was significantly more oxidized when H_2_O_2_ treatment followed SI induction, compared to H_2_O_2_ treatment alone (Fig. S4c). These results suggest that the increases in roGFP2-Orp1 oxidation in the cytosol and mitochondria after SI induction are due to elevated H_2_O_2_ production. In contrast, the rise in roGFP2-Orp1 oxidation in plastids might be partly due to a compromised plastid reduction capacity following SI induction.

### The electron transport chain contributes to SI-induced H_2_O_2_ elevation in mitochondria

**Figure 4:**
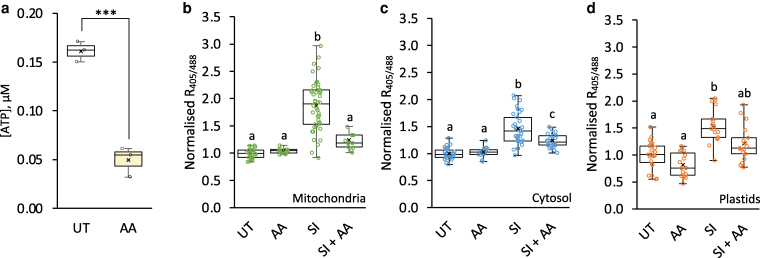
Inhibition of the electron transport chain (ETC) mitigates SI-induced ROS elevation in Arabidopsis pollen tubes. **a)** Quantification of ATP levels in untreated (UT) pollen tubes or 20 min after the addition of 10 μM antimycin A (AA). ATP levels were significantly reduced following treatment (***P < 0.001, one-way ANOVA; n = 3). **b-d)** Quantification of 405/488 nm fluorescence ratio (R_405/488_) of roGFP2-Orp1 targeted to mitochondria **(b)**, cytosol **(c)**, and plastids **(d)** in untreated (UT) pollen tubes or 20 min after treatment with AA, with or without SI induction. n ≥ 13. Different letters indicate significant (*P* < 0.001) differences based on Tukey's test. The box area of the boxplots contains the central 50% of values (first quartile to third quartile), whiskers indicate 1.5 times the interquartile range. The median is represented by a horizontal line, and the mean is indicated by a cross. Circles represent individual data points.

Mitochondrial ROS are generated as a by-product of the electron transport chain (ETC) during respiration. To investigate the contribution of the ETC to H_2_O_2_ production during SI in the pollen tube, we used antimycin A, an inhibitor of complex III, to disrupt the ETC during SI responses (Wagner et al. 2019 ; Rattanawong et al. 2021). Treatment of growing pollen tubes with antimycin A for 20 min resulted in ATP depletion (Fig. 4a) but did not significantly affect roGFP2-Orp1 oxidation state in the cytosol and mitochondria of growing pollen tubes (Fig. 4b and c). Importantly, antimycin A mitigated the SI-induced increases in roGFP2-Orp1 oxidation in mitochondria and the cytosol after 20 min of SI induction (Fig. 4b and c). This supports the hypothesis that the ETC contributes to SI-induced increases in H_2_O_2_ production in mitochondria and that this mitochondrial H_2_O_2_ can diffuse into the cytosol. A similar trend was observed for plastids, where SI-induced oxidation was partially (although not significantly) reduced by antimycin A (Fig. 4d). Consistent with ETC inhibition and mitochondrial dysfunction, antimycin A also caused a rapid decrease in the mitochondrial membrane potential within 5 min of treatment (Fig. S5).

### SI and H_2_O_2_ induce the collapse of mitochondrial membrane potential (Δψ_m_)

**Figure 5:**
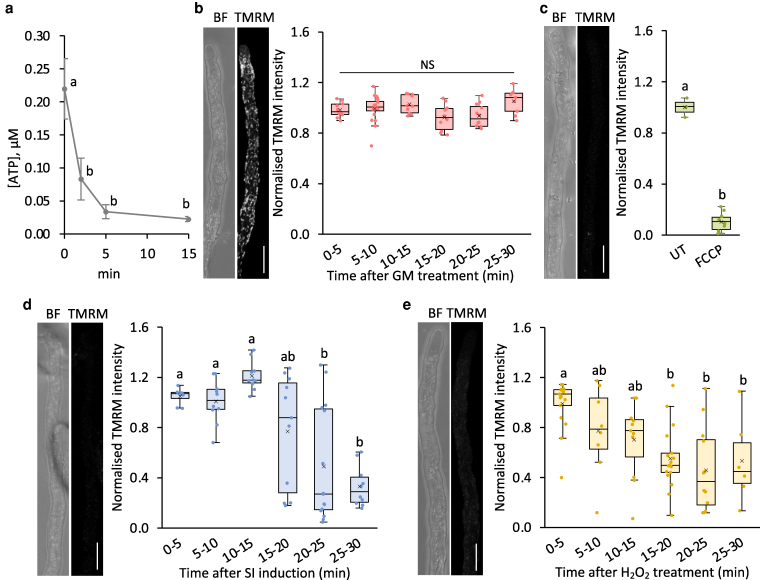
SI and H_2_O_2_ induce decreases in mitochondrial membrane potential (ΔΨ_m_) in Arabidopsis pollen tubes. **a)** Quantification of ATP levels in pollen tubes treated with 2.5 mM H_2_O_2_. **b-e)** Left panels show representative bright field (BF) and TMRM fluorescence images of pollen tubes after 20 min **(b–d)** or 10 min **(e)** of treatment. Scale bar = 10 μm. Right panels show the corresponding quantification of TMRM fluorescence in mitochondria after treatment with growth medium (GM) **(b)**, FCCP **(c)**, SI induction **(d)**, and treatment with 2.5 mM H_2_O_2_ **(e)**. Different letters indicate significant differences based on Tukey's test **(a** and **c**, *P* < 0.001; **d**, *P* < 0.05; **e**, *P* < 0.01). Samples sizes: **a**, n = 3; **b**, n = 9 to 20; **c**, n = 3 to 10; **d**, n = 8 to 13; **e**, n = 6 to 17. The box area of the boxplots contains the central 50% of values (first quartile to third quartile), whiskers indicate 1.5 times the interquartile range. The median is represented by a horizontal line, and the mean is indicated by a cross. Circles represent individual data points.

As we previously showed that SI induces ATP depletion within just a few minutes (Wang et al. 2022), we next investigated whether mitochondrial dysfunction could contribute to this rapid energy loss. Treatment of pollen tubes with H_2_O_2_ resulted in rapid decreases in cellular ATP levels, falling to ∼38% of the original level within 2 min, and to ∼10% by 15 min (Fig. 5a), suggesting that elevated ROS can directly compromise energy status. As Δψ_m_ is essential for ATP synthase function, we assessed whether SI and H_2_O_2_ also affect Δψ_m_.

To measure Δψ_m_ in pollen tubes, we used the well-established Δψ_m_ probe, tetramethyl rhodamine methyl ester (TMRM) (Perry et al. 2011), along with carbonyl cyanide-p-trifluoromethoxyphenylhydrazone (FCCP), a potent uncoupler of Δψ_m_ (Khan et al. 2024). In growing pollen tubes, a bright, stable TMRM signal was detected, indicating a healthy mitochondrial ΔΨ_m_ (Fig. 5b). Treatment with FCCP for 10 min resulted in a significantly diminished TMRM signal (Fig. 5c), confirming the probe's responsiveness to changes in Δψ_m_. After SI induction, TMRM fluorescence was significantly reduced within 15 to 20 min, to 77.2 ± 45.0% of the initial level, with a further decrease to 33.1 ± 16.2% by 25 to 30 min (Fig. 5d), demonstrating collapse of ΔΨ_m_. Similarly, treatment with 2.5 mM H_2_O_2_ caused TMRM fluorescence to decrease to 55.3% by 15 to 20 min, remaining relatively stable thereafter (Fig. 5e). This demonstrates that both SI and H_2_O_2_ induce collapse of ΔΨ_m_. These results, together with previous reports of cytochrome *c* release and mitochondrial swelling in incompatible *Papaver* pollen tubes, indicate that Δψ_m_ collapse represents part of a broader decline in mitochondrial integrity triggered by SI.

### Manipulation of [Ca^2+^]_cyt_ alters SI-induced collapse of Δψ_m_ and roGFP2-Orp1 oxidation

**Figure 6:**
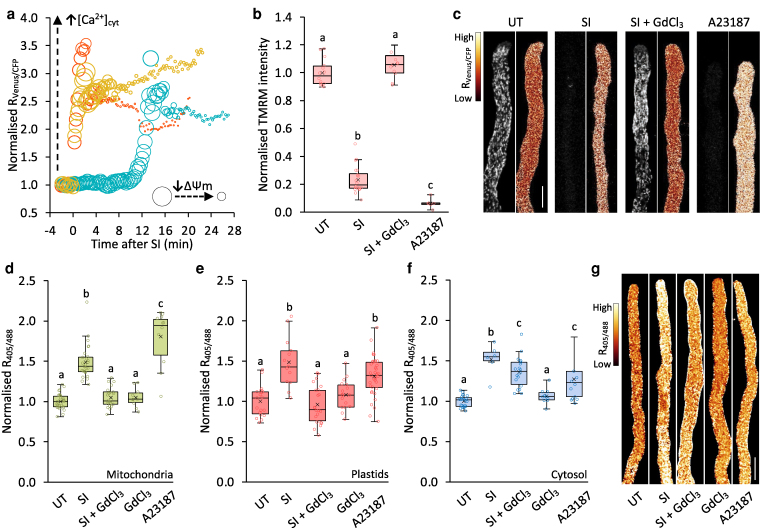
SI-induced roGFP2-Orp1 oxidation and mitochondrial ΔΨ_m_ are dependent on increases in [Ca^2+^]_cyt_. **a)** Quantification of YC3.6 fluorescence ratio (R_Venus/CFP_) and TMRM fluorescence intensity in three representative Arabidopsis pollen tubes expressing PrpS_1_ and YC3.6 following treatment with PrsS_1_ (SI induction). A higher R_Venus/CFP_ indicates increased [Ca^2+^]_cyt_. Smaller bubble widths indicate lower TMRM fluorescence intensity, which reflects a decrease in ΔΨ_m_. Data from individual pollen tubes are shown in gold, red, and teal. **b)** Quantification of TMRM fluorescence intensity in mitochondria in untreated (UT) pollen tubes and at 25 to 40 min after treatment with PrsS_1_ (SI), PrsS_1_ plus GdCl_3,_ or A23187. n = 14, 20, 22, 6, respectively. **c)** Representative images show YC3.6 fluorescence ratio (R_Venus/CFP_; right panels) and TMRM fluorescence signal (left panels) in untreated (UT) pollen tubes and 20 min after treatment with PrsS_1_ (SI), PrsS_1_ plus GdCl_3,_ or A23187. Scale Bar, 10 μm. **d-f)** Quantification of 405/488 nm fluorescence ratio (R_405/488_) of roGFP2-Orp1 targeted to mitochondria **(d)**, plastids **(e)**, and cytosol **(f)** in untreated (UT) pollen tubes or 20 min after treatment with PrsS_1_ (SI), PrsS_1_ plus GdCl_3_, GdCl_3_ only, or A23187. Sample sizes: **d**, n = 10 to 30; e, n = 13 to 36; f, n = 9 to 22, respectively. **g)** Representative ratio images of pollen tubes expressing PrpS_1_ and roGFP2-Orp1 under the same conditions as in **(f)**. Scale bar, 10 μm. In **b**, **d**, **e**, and **f**, different letters indicate significant (*P* < 0.05) differences based on Tukey's test. The box area of the boxplots contains the central 50% of values (first quartile to third quartile), whiskers indicate 1.5 times the interquartile range. The median is represented by a horizontal line, and the mean is indicated by a cross. Circles represent individual data points.

[Ca^2+^]_cyt_ dynamics are important for mitochondrial calcium homeostasis and influence Δψ_m_ (Duchen 2000; Loro et al. 2012). To investigate the relationship between SI-induced increases in [Ca^2+^]_cyt_ and collapse of ΔΨ_m_, we simultaneously imaged [Ca^2+^]_cyt_ and ΔΨ_m_ in pollen tubes using the genetically encoded [Ca^2+^]_cyt_ indicator YC3.6 and the TMRM probe. Tracking individual pollen tubes revealed that increases in YC3.6 R_venus/CFP_ ratios occurred before decreases in TMRM fluorescence (Fig. 6a). Although the precise timing varied between individual pollen tubes, in all cases the [Ca^2+^]_cyt_ elevation preceded the decline in ΔΨ_m_. This suggests that the SI-induced [Ca^2+^]_cyt_ elevation is an upstream event leading to the collapse of Δψ_m_. To further explore this link, we compared the effects of SI induction and calcium ionophore (A23187) treatment, in combination with the calcium channel blocker GdCl_3_. Both SI and A23187 treatments induced substantial increases in [Ca^2+^]_cyt_ (Fig. 6c; Fig. S6) and led to substantial decreases in TMRM fluorescence intensity after 25 to 40 min compared to untreated pollen tubes (Fig. 6b). However, GdCl_3_ maintained TMRM fluorescence intensity in SI-induced pollen tubes at the same level as untreated pollen tubes (105.7 ± 8.0%, *P* = 0.23; Fig. 6b and c), confirming that Ca^2+^ influx is required for the SI-induced collapse in ΔΨ_m_.

To examine possible links between [Ca^2+^]_cyt_ and ROS in mitochondria, plastids, and the cytosol, we quantified roGFP2-Orp1 oxidation state in PrpS1-expressing pollen tubes treated with A23187 and GdCl_3_. In mitochondria and plastids, both SI induction and A23187 treatment significantly increased roGFP2-Orp1 oxidation (Fig. 6d and e). SI induction in the presence of GdCl_3_ prevented the SI-induced roGFP2-Orp1 oxidation in both mitochondria and plastids; the R_405/488_ ratios in these compartments were not significantly different from untreated pollen tubes (*P* = 0.84 for mitochondria and *P* = 0.98 for plastids; Fig. 6d and e). In the cytosol, the effect of GdCl_3_ on SI-induced roGFP2-Orp1 oxidation was less pronounced than in the mitochondria and plastids (Fig. 6f). Pollen tubes displayed elevated roGFP2-Orp1 oxidation (R_405/488_) at 20 min after SI induction (1.67 ± 0.28-fold, *P* < 0.001) or A23187 treatment (1.27 ± 0.25, *P* < 0.001). Co-treatment with GdCl_3_ significantly impaired SI-induced roGFP2-Orp1 oxidation compared to SI alone (*P* < 0.001; Fig. 6f and g). Together, these data show that Ca^2+^ influx and increases in [Ca^2+^]_cyt_ are required to achieve elevation of SI-induced ROS across these compartments, implicating Ca^2+^ signaling upstream of ROS increases.

### Manipulation of cytosolic pH modulates SI-induced decrease in Δψ_m_

**Figure 7:**
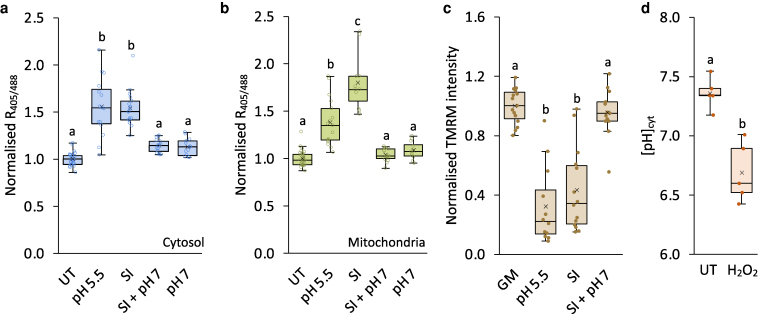
Manipulation of cytosolic pH modulates SI-induced mitochondrial dysfunction in Arabidopsis pollen tubes. **a, b)** Quantification of 405/488 nm fluorescence ratio (R_405/488_) of roGFP2-Orp1 targeted to the cytosol **(a)** and mitochondria **(b)** in untreated (UT) pollen tubes and at 20 min following treatment with propionic acid at pH 5.5, PrsS_1_ (SI), PrsS_1_ with propionic acid at pH 7 (SI + pH 7), or propionic acid at pH 7 alone. n ≥ 11. **c)** Quantification of TMRM fluorescence intensity in mitochondria of pollen tubes treated with growth medium (GM), PrsS_1_ (SI), PrsS_1_ with propionic acid at pH 7 (SI + pH 7), or propionic acid at pH 5.5 for 25 mins. Treatment at pH 5.5 triggered decreases in mitochondrial ΔΨ_m_ similar to those induced by SI, while clamping at pH 7.0 prevented SI-induced decreases in mitochondrial ΔΨ_m_. n ≥ 12. **d)** Quantification of cytosolic pH ([pH]_cyt_) in pollen tubes 25 min following treatment with 2.5 mM H_2_O_2_. n = 5. Different letters in a-d indicate significant (*P* < 0.001) differences based on Tukey's test. The box area of the boxplots contains the central 50% of values (first quartile to third quartile), whiskers indicate 1.5 times the interquartile range. The median is represented by a horizontal line, and the mean is indicated by a cross. Circles represent individual data points.

Since SI also triggers cytosolic acidification (Wilkins et al. 2015; Wang et al. 2022), we investigated whether changes in [pH]_cyt_ affect alterations in the oxidation state of roGFP2-Orp1 in the cytosol and mitochondria. Artificially reducing [pH]_cyt_ to 5.5 using propionic acid significantly increased roGFP2-Orp1 oxidation (R_405/488_) in both the cytosol (Fig. 7a) and mitochondria (Fig. 7b) compared to untreated pollen tubes. Conversely, clamping the [pH]_cyt_ at 7 with propionic acid prevented SI-induced roGFP2-Orp1 oxidation in both the cytosol (Fig. 7a) and mitochondria (Fig. 7b) compared to untreated pollen tubes. These treatments also affected ΔΨ_m_ (Fig. 7c). SI induction and lowering [pH]_cyt_ to 5.5 decreased TMRM fluorescence intensity. In contrast, clamping [pH]_cyt_ at 7 prevented SI-induced decreases in TMRM fluorescence. These findings firmly implicate cytosolic acidification in the signaling network underlying these processes. To assess whether H_2_O_2_ participates in a feedback loop affecting [pH]_cyt_, we treated pollen tubes with 2.5 mM H_2_O_2_, which decreased [pH]_cyt_ to 6.69 compared to untreated pollen tubes with a [pH]_cyt_ of 7.28 (Fig. 7d), though the acidification was less pronounced than that induced by SI, which we have previously shown reduced [pH]_cyt_ to ∼5.5 in both *Papaver* and engineered Arabidopsis pollen tubes (Wilkins et al. 2015; Wang et al. 2020, 2022). Together, these results indicate that cytosolic acidification contributes to SI-induced mitochondrial dysfunction and H_2_O_2_ generation.

### SI and H_2_O_2_ inhibit respiratory metabolism in pollen tubes

We previously identified irreversible oxidation of several enzymes associated with glycolysis, organic acid metabolism, and mitochondrial ATP synthase that were induced by both SI and H_2_O_2_ (Haque et al. 2020). As SI triggers rapid ATP depletion, this suggests significant energetic and metabolic changes occur in incompatible pollen tubes (Wang et al. 2022). Our data indicate that SI triggers increased mitochondrial H_2_O_2_ generation and decreased mitochondrial membrane potential. To explore the impact of SI on respiratory metabolism, we measured oxygen uptake, the activity of the glycolytic enzyme glyceraldehyde 3-phosphate dehydrogenase (GAPDH), and the concentration of TCA cycle intermediates in response to SI and H_2_O_2_.

**Figure 8:**
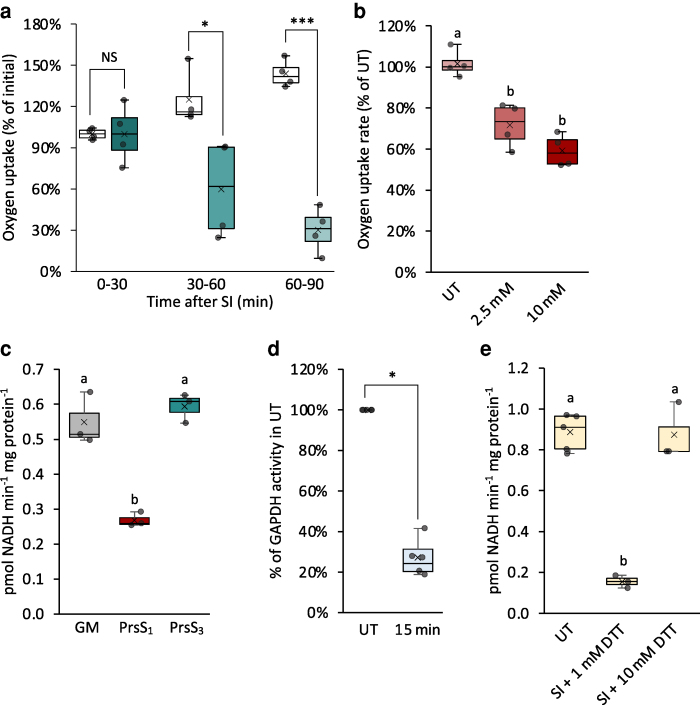
SI and H_2_O_2_ inhibit glycolysis and mitochondrial respiration in pollen tubes. **a)** Oxygen uptake rates in *Papaver* pollen tubes during three time intervals: 0 to 30 min, 30 to 60 min, and 60 to 90 min after treatment with growth medium (control, empty bars) or after SI induction (filled bars). Rates were normalized to mean values during the 0 to 30 min interval. NS, not significant. *P < 0.05, ***P < 0.001, one-way ANOVA. n = 4. **b)** Oxygen uptake rates in untreated (UT) *Papaver* pollen tubes and those treated with 2.5 mM or 10 mM H_2_O_2_ for 30 min. Rates were normalized to mean values of untreated samples. (n = 4). **c)** GAPDH activities in Arabidopsis pollen tubes measured 5 min after treatment with growth medium (GM), PrsS_1_ (SI induction), or PrsS_3_ (n = 3). **d)** GAPDH activities in Arabidopsis pollen tubes 15 min after treatment with 2.5 mM H_2_O_2_. *P < 0.05, Mann–Whitney U test (n = 4). **e)** DTT prevented SI-induced inactivation of GAPDH in Arabidopsis pollen tubes. n ≥ 3. **b, c,** and **e)**, Different letters indicate significant differences based on Tukey's test **(b,** *P* < 0.01, **c** and **e,** *P* < 0.001). The box area of the boxplots contains the central 50% of values (first quartile to third quartile), whiskers indicate 1.5 times the interquartile range. The median is represented by a horizontal line, and the mean is indicated by a cross. Circles represent individual data points.

To assess whether SI and ROS inhibit respiration, we measured the oxygen consumption of *Papaver* pollen tubes. Using *Papaver* pollen tubes was necessary due to the limited amount of Arabidopsis pollen available, so direct comparison of time courses with Arabidopsis is not possible. Following SI induction, oxygen uptake rates significantly decreased, dropping to 59.7% at 30 to 60 min and to 30% at 60 to 90 min compared to growth medium treated controls (Fig. 8a). Following treatment with 2.5 and 10 mM H_2_O_2_, oxygen uptake rates decreased to 71.6% and 59.2%, respectively, during the 30 to 60 min period compared to untreated samples (Fig. 8b).

**Figure 9:**
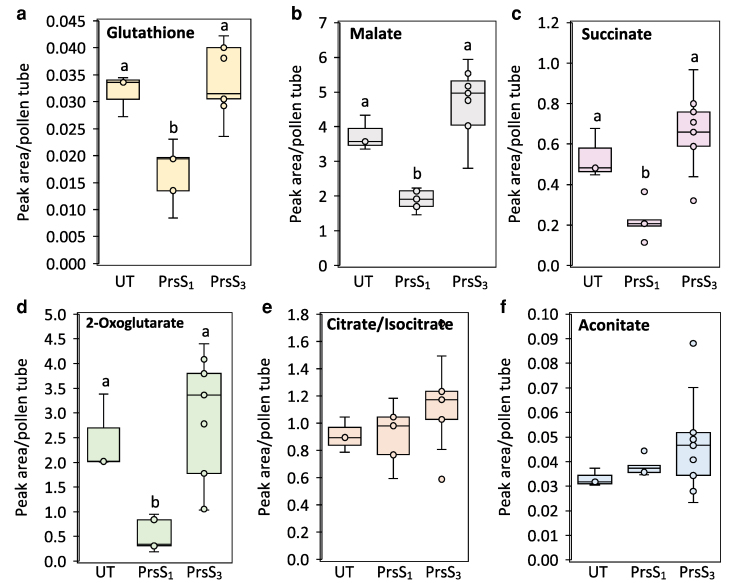
SI induces decreases some TCA cycle acids and glutathione in Arabidopsis pollen tubes. Levels of glutathione **(a)**, malate **(b)**, succinate **(c)**, and 2-oxoglutarate **(d)** were significantly reduced in pollen tubes 15 min after SI induction. In contrast, levels of citrate/isocitrate **(e)** and aconitate **(f)** remained unchanged. Sample sizes: Untreated (UT), n = 3; PrsS_1_, n = 5; PrsS_3_, n = 9. Different letters indicate significant (*P* < 0.001) differences based on Tukey's test. The box area of the boxplots contains the central 50% of values (first quartile to third quartile), whiskers indicate 1.5 times the interquartile range. The median is represented by a horizontal line, and the mean is indicated by a cross. Circles represent individual data points.

As GAPDH was previously identified as a target for oxidative post-translational modifications in *Papaver* pollen tubes triggered by both SI and H_2_O_2_ (Haque et al. 2020), we measured its activity in Arabidopsis pollen extracts. 5 min after SI induction, GAPDH activity was significantly reduced to 48.9% of the activity in control samples. This effect was SI-specific, as compatible treatment had no significant effect on GAPDH activity relative to control (GM) samples (Fig. 8c). This demonstrates that SI induces rapid impairment of GAPDH activity in incompatible pollen tubes. Treatment of pollen tubes with H_2_O_2_ also led to a decrease in GAPDH activity. Within 15 min of treatment, GAPDH activity was reduced to 27% of the level in untreated pollen tubes (Fig. 8d). As addition of 10 mM dithiothreitol (DTT) restored GAPDH activity (Fig. 8e), this suggests that SI and H_2_O_2_ inactivate GAPDH by oxidation of cysteine thiol groups to sulfenic acids, while further oxidation of the sulfenic acid gives rise to the previously reported oxidative post-translational modifications (Haque et al. 2020). Decreased GAPDH activity following SI induction or H_2_O_2_ treatment was also detected in *Papaver* pollen tubes (Fig. S7). These findings suggest that SI-induced inactivation of GAPDH is mediated by elevated levels of ROS stimulated by SI. Further, glutathione (GSH), a prominent component of the thiol antioxidant system, decreased within 15 min of SI (Fig. 9a), supporting the occurrence of wider redox perturbation. This suggests that glycolysis and the supply of pyruvate to the TCA cycle are rapidly decreased during SI. Consistent with this conclusion, the concentration of 2-oxoglutarate, succinate,e and malate had decreased by ∼50% within 15 min of initiating SI with PrsS_1_ but not in the compatible response with PrsS_3_ (Fig. 9b-d). In a separate experiment, 2-oxoglutarate decreased within 15 mins, while malate and succinate showed a progressive (but not significant) decrease from 15 to 120 min post SI induction (Fig. S8). Intriguingly, the TCA cycle acids at the beginning of the cycle (citrate/isocitrate and aconitate) were unaffected (Fig. 9e and f and Fig. S8c). These findings demonstrate that both SI and elevated H_2_O_2_ disrupt key metabolic pathways, rapidly impairing energy production and redox homeostasis in pollen tubes.

## Discussion

**Figure 10:**
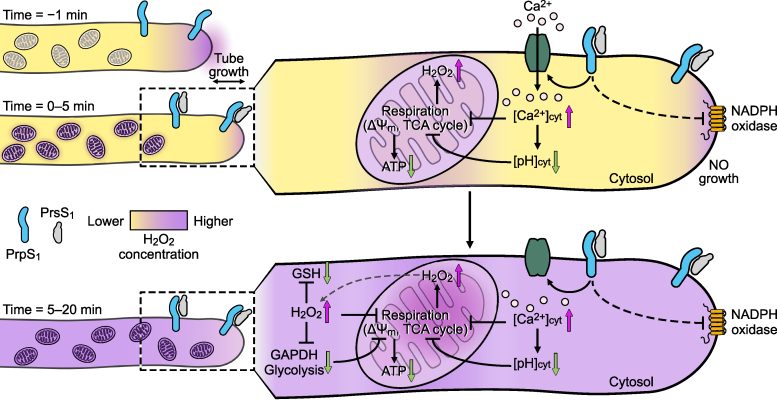
A summary of the interconnected events occurring during the first 10 to 20 min of the SI response in incompatible pollen tubes. We previously showed that SI is initiated when the stigmatic *S*-determinant PrsS (PrsS_1_ in the diagram) binds to its cognate pollen plasma membrane protein PrpS_1_. This triggers a rapid influx of Ca^2+^, resulting in a transient of elevated cytosolic Ca^2+^ concentration ([Ca^2+^]_cyt_), disruption of the tip-focused Ca^2+^ gradient, and arrest of pollen tube tip growth within 1 to 2 min. Downstream of the [Ca^2+^]_cyt_ increases, a network of intracellular responses is rapidly triggered, including cytosolic acidification and decreased ATP concentration. The cartoon depicts events identified in this study in incompatible pollen tubes over three time periods: before SI (−1 min), 0 to 5, and 5 to 20 min after initiation of SI. The larger cartoons on the right indicate key changes observed in this study and a model of how this may operate to mediate SI. **0 to 5 min after SI.** Tip-localized NADPH oxidase is inactivated, resulting in the inhibition of apical ROS production and arrest of tip growth. SI triggers increased roGFP2-Orp1 oxidation in the cytosol and mitochondria. Mitochondria are the primary source of SI-induced increases in H_2_O_2_ (indicated in purple), and H_2_O_2_ generated here diffuses into the cytosol. This demonstrates that spatially and temporally distinct ROS signals can serve as intracellular signatures that allow for functionally divergent roles within the pollen tube. Mitochondrial membrane potential (ΔΨ_m_) decreases, concomitant with decreased ATP concentration. As manipulation of cytosolic pH ([pH]_cyt_) shows that acidification is sufficient to decrease ΔΨ_m_ and elevate H_2_O_2_, and maintaining neutral [pH]_cyt_ prevents these effects, this suggests that cytosolic acidification acts as an early effector in this SI response. **5 to 20 min after SI.** The H_2_O_2_ generated by mitochondria diffuses into the cytosol, leading to large increases in cytosolic H_2_O_2_ (purple). SI also results in reduced levels of antioxidants such as glutathione (GSH), demonstrating that redox homeostasis is further compromised. In addition, SI triggers oxidative inactivation of glyceraldehyde 3-phosphate dehydrogenase (GAPDH), a glycolytic enzyme. This will limit glycolytic flux and reduce pyruvate availability for mitochondrial respiration, contributing to decreased tricarboxylic acid (TCA) cycle activity, thereby reducing ATP production in incompatible pollen tubes further. These findings provide insights into how redox signaling can modulate energy metabolism. Decreased ATP may itself exacerbate cytosolic acidification *via* inhibition of ATP-dependent H⁺-ATPases at the plasma membrane and/or vacuole. We propose a model whereby these early SI-induced events establish a positive feedback loop in which SI-induced Ca^2+^ and pH changes disrupt mitochondrial function, which in turn generates further ROS and suppresses energy metabolism, amplifying intracellular disruption. *Color and symbol key:* Intensity of purple color indicates H_2_O_2_ concentration. Black arrows denote activation/increases, black flat-end arrows denote inhibition. Pink and green arrows indicate increases and decreases of components compared to the state prior to SI initiation. *Abbreviations*: ΔΨ_m_, mitochondrial membrane potential; TCA, tricarboxylic acid cycle; GSH, glutathione; GAPDH, glyceraldehyde 3-phosphate dehydrogenase; [Ca^2+^]_cyt_, cytosolic calcium concentration; [pH]_cyt_, cytosolic pH.

Using the heterologous Arabidopsis system, which exhibits key features of *Papaver* SI (Lin et al. 2020; Wang et al. 2020), we provide an integrated, subcellular-resolution analysis of the early SI response in pollen. This has revealed that the SI response disrupts mitochondrial electron transport, triggers compartment-specific H_2_O_2_ accumulation, and suppresses glycolytic flux *via* oxidation-dependent inactivation of GAPDH. These interconnected events are summarized in Fig. 10. Previously, we showed that SI in *Papaver* stimulates rapid ROS production and H_2_O_2_-dependent protein oxidation in incompatible pollen tubes (Wilkins et al. 2011; Haque et al. 2020). Here, using the genetically encoded H_2_O_2_ biosensor roGFP2-Orp1 (Gutscher et al. 2009; Nietzel et al. 2019; Ugalde et al. 2021; Arnaud et al. 2023), we dissected H_2_O_2_ dynamics across five subcellular compartments, identifying mitochondria as the primary site of SI-induced ROS generation and the likely origin of H_2_O_2_ increases in the cytosol and plastids. Importantly, we also show that the ROS required for pollen tube growth, produced *via* NADPH oxidase activity at the tip, is functionally and spatially distinct from the SI-induced ROS. This multi-layered disruption occurs within 20 min of SI induction and is orchestrated through Ca^2+^ influx and a subsequent rise in [Ca^2+^]_cyt,_ followed soon after by cytosolic acidification, mitochondrial membrane depolarization, ATP depletion, and redox perturbation. Pharmacological and biosensor data suggest that mitochondrial dysfunction is both a source and a target of redox and pH perturbations, setting up a feedback loop that accelerates cellular shutdown. Thus, this study provides a mechanistic framework that links early signal transduction initiated by SI to rapid metabolic failure and oxidative stress within the first 10 to 20 min, which is well before PCD execution.

### SI-induced ROS is distinct from NADPH oxidase-sourced ROS necessary for tip growth

The NADPH oxidase isoforms RBOHH and RBOHJ, localized at the plasma membrane of the pollen tube tip, are required for pollen tube growth in Arabidopsis. Pollen tubes of mutants lacking both isoforms (*rbohH/J*) are prone to bursting and fail to reach the female gametophyte (Boisson-Dernier et al. 2013; Kaya et al. 2014). NADPH oxidase forms superoxide in the apoplast, which is rapidly dismutated to H_2_O_2_. Apoplastic NADPH oxidase-dependent superoxide production at the pollen tube tip has previously been detected by NBT reduction in tobacco (Potocký et al. 2007) and decreased oxidation of cytosolic HyPer, an H_2_O_2_ biosensor (Boisson-Dernier et al. 2013). We confirm this in both *Papaver* and Arabidopsis because, as anticipated, SI caused a very rapid loss of tip-localized superoxide production which coincided with tip growth arrest. NADPH oxidase activity is subject to complex control (Potocký et al. 2003, 2012) and inhibition of NADPH oxidase by the Ca^2+^ channel blocker Gd^3+^, confirmed a role for Ca^2+^ influx in its activation. The rapid SI-induced inactivation of NADPH oxidase is most likely due to disruption of the normal [Ca^2+^]_cyt_ signature of tip-high, oscillating [Ca^2+^]_cyt_ that is required for growth (Franklin-Tong et al. 1997). Our results demonstrate that the ROS required for normal pollen tube tip growth, originating from NADPH oxidase activity, are functionally distinct from the mitochondrially produced ROS induced by SI (Fig. 10).

### Mitochondria are the source of the SI-induced H_2_O_2_ production

To track intracellular H_2_O_2_ dynamics during the SI response, we used roGFP2-Orp1 targeted to various organelles of Arabidopsis pollen co-expressing PrpS. The biosensor was functional, responding as expected to both H_2_O_2_ and the reductant dithiothreitol. Importantly, roGFP2-Orp1 remains functional at pH 5.5 (Nietzel et al. 2019), so the measured oxidation state is unaffected by the major cytosolic acidification induced by SI or treatment with propionic acid. Initiation of SI caused oxidation of the biosensor in the cytosol, mitochondria, and plastids, but not in the nuclei and peroxisomes. Since roGFP2-Orp1 reports the redox balance between oxidation by H_2_O_2_ and its reduction by the thiol system, the signal is to some extent a reflection of both processes (Nietzel et al. 2019). The kinetics of oxidation were very similar in each compartment, with oxidation becoming evident after ∼10 min. This is like the time course of CM-H_2_DCF oxidation previously observed after triggering SI in *Papaver* (Wilkins et al. 2011), but the use of roGFP2-Orp1 has allowed us to establish that this involves H_2_O_2_ and also enabled the subcellular location of oxidation to be established. Since H_2_O_2_ can diffuse across membranes, facilitated by aquaporins (Bienert and Chaumont 2014; Rodrigues et al. 2017), its source is not immediately obvious. However, the mitochondrial electron transport inhibitor antimycin A, which inhibits complex III, prevented SI-induced roGFP2-Orp1 oxidation in mitochondria and cytosol. This suggests that SI-induced H_2_O_2_ production is dependent on mitochondrial electron transport. Although it is reported that antimycin A can increase ROS production, the effect is not always large (Maxwell et al. 1999; Møller 2001) nor consistent. For example, antimycin A treatment of Arabidopsis seedlings did not result in rapid oxidation of cytosolic roGFP2-Orp1 (Khan et al. 2024), but in pollen, SI-induced oxidation is rapid and is inhibited by antimycin A across mitochondria and cytosol. Thus, our results strongly suggest that mitochondria are the major source of SI-induced H_2_O_2_ (Fig. 10), likely *via* residual electron transport, with ROS subsequently diffusing into the cytosol and plastids. It is also possible that the decreased cytosolic pH inhibits H_2_O_2_ scavenging enzymes such as ascorbate peroxidase and could contribute to SI-induced H_2_O_2_ accumulation. In particular, the plastid form of ascorbate peroxidase has a steep decrease of activity below its pH 8.0 optimum, but the cytosolic form maintains activity to pH 4.5 (Mittler and Zilinskas 1991). Investigation of the pH response of all possible H_2_O_2_ removing enzymes would be intractable.

Overall, the results are consistent with SI causing a disruption in mitochondrial metabolism that results in oxygen reduction to superoxide and consequent H_2_O_2_ production. We postulate that H_2_O_2_ rapidly moves to the cytosol and plastids. The rapid oxidation observed in plastids is unlikely to result from ROS generation within the organelle itself, since plastids do not have an electron transport chain (Renato et al. 2015). Instead, plastid ROS accumulation most likely reflects diffusion of H_2_O_2_ from mitochondria, potentially *via* aquaporins. As plastids support rapid pollen tube growth *via* glycolysis, the oxidative pentose phosphate pathway, and fatty acid synthesis (Emes and Neuhaus 1997), their redox state may be affected by SI-induced growth arrest. The lack of a response in peroxisomes may reflect their specialization in housing H_2_O_2_-producing oxidases along with high catalase activity (Smirnoff and Arnaud 2019).

Our results provide a clear example of a ROS signature: two spatially distinct ROS sources playing divergent functional roles within a single cell type, the vegetative cell of the pollen tube, which contains the generative and vegetative nuclei. While the concept and evidence for distinct spatial-temporal Ca^2+^ “signatures” are well-established (Lecourieux et al. 2006; McAinsh and Pittman 2009; Monshausen 2012), the question of how ROS signals can generate specific responses has been discussed for some time (Mittler et al. 2022). ROS originating from different subcellular compartments following different stimuli can bring about distinct responses (Torres et al. 2002; Foreman et al. 2003; Potocký et al. 2007; Van Aken and Van Breusegem 2015; Exposito-Rodriguez et al. 2017; Page et al. 2017; Arnaud et al. 2023; Postiglione and Muday 2023; Khan et al. 2024). Together, these findings support a model in which the timing, localization, and source of ROS generation are critical in determining specific physiological outcomes, even within a single cell. In the future, further use and development of genetically encoded tools to visualize spatio-temporal patterns of different ROS should allow further dissection and elucidation of ROS signatures in response to a variety of biologically relevant stimuli.

### SI decreases glycolysis by oxidative inactivation of GAPDH and inhibits the TCA cycle

SI and exogenous H_2_O_2_ caused a decrease in oxygen uptake by *Papaver* pollen, demonstrating that SI has a large impact on respiration. Several lines of evidence point to disrupted metabolism, including decreased ATP, reduced GAPDH activity (affecting glycolysis in both cytosol and plastids), a decrease in some TCA cycle intermediates, and a decrease in the mitochondrial membrane potential (Fig. 10). We previously identified redox-sensitive post-translational modifications in two glycolytic enzymes, GAPDH and enolase, following both SI and H_2_O_2_ treatment in *Papaver* pollen (Haque et al. 2020), implicating oxidative damage to these proteins. Here, we show that some of these modifications have functional significance since we found that NAD-GAPDH activity is rapidly decreased during SI and following H_2_O_2_ treatment. Crucially, this inactivation was reversed by DTT, indicating that reversible cysteine oxidation is involved. GAPDH is well known to be under redox control, undergoing reversible oxidation by H_2_O_2_ on a conserved cysteine residue (Cys155), resulting in inactivation (Schneider et al. 2018; Dumont and Rivoal 2019). As well as influencing glycolysis rate in the cytosol and plastids, the oxidized form of GADPH can translocate to the nucleus and may be involved in redox signaling, while the reduced form associates with voltage-dependent anion channels (VDAC) on the outer mitochondrial membrane, possibly as part of a glycolytic complex (Dumont and Rivoal 2019). The oxidative inactivation of glycolysis will impose a limitation on ATP generation by substrate-level phosphorylation and decrease the supply of pyruvate to the mitochondria. The latter will limit TCA cycle activity, as shown by the decrease in TCA cycle intermediates beyond citrate/isocitrate. Moreover, given that SI triggers a decrease in glycolysis, the possibility of ATP generation by fermentation, as suggested by Wang et al. (2022), is unlikely. This conclusion is reinforced for Arabidopsis, whose pollen has a low fermentation capacity (Liu et al. 2022). In support of an early impact on metabolism, previous work in *Papaver* has shown that soluble inorganic pyrophosphatases (sPPases), which are essential for biosynthetic reactions, become phosphorylated and inactivated within minutes of SI induction (Rudd et al. 1996; de Graff et al. 2006), further illustrating the rapid shutdown of key metabolic processes following SI. Together, our data establish a direct mechanistic link between SI-induced ROS and impaired metabolism *via* redox regulation of GAPDH (Fig. 10) and suggest that oxidative modifications of other glycolytic enzymes may also contribute. While some studies, such as Obermeyer et al. (2013), have inferred changes in pollen metabolism in response to mitochondrial dysfunction using proteomic data, to date, direct evidence for ROS-mediated inactivation remains scarce in plants. These findings therefore provide important insights into how redox signaling can modulate energy metabolism in response to cellular stress signals such as SI.

### SI triggers multi-layered cellular disruption.

SI induction very rapidly triggers H_2_O_2_ production and disruption to respiratory metabolism. The previously described increases in [Ca^2+^]_cyt_, cytosolic acidification, and decrease in ATP levels are equally rapid, making it difficult to unravel cause and effect (Wilkins et al. 2011, 2015; Wang et al. 2019, 2022; Haque et al. 2020). However, integrating our findings enables us to propose a model outlining the likely sequence and interdependence of these early events during the first 10 to 20 min of the SI response (Fig. 10). Elevated [Ca^2+^]_cyt_ is the earliest detectable change (Bosch and Franklin-Tong 2024). Pharmacological manipulation using Ca^2+^ channel blockers and ionophores indicates that several key events, including a decrease in ΔΨ_m_, increased roGFP2-Orp1 oxidation (reflecting H_2_O_2_ production), decreased ATP levels, and cytosolic acidification, are downstream of the initial influx and subsequent increases in [Ca^2+^]_cyt_. As [Ca^2+^]_cyt_ increase causes an increase in mitochondrial matrix Ca^2+^ (Ruberti et al. 2022 and references therein), it is likely that impairment of mitochondrial function in SI is due to mitochondrial matrix “Ca^2+^ overload” and a collapse of function (Walkon et al. 2022). As overload increases, mitochondria may even consume ATP rather than synthesize it (Walkon et al. 2022). Whether SI causes increased Ca^2+^ uptake by mitochondria should now be tested.

Furthermore, manipulation of [pH]_cyt_ shows that acidification increases H_2_O_2_ and decreases ΔΨ_m,_ while these SI-induced events are blocked by maintaining neutral [pH]_cyt_. Acidification may therefore act as an early effector in the SI response, showing that cytosolic acidification can impair mitochondrial function in plants. While the importance of pH homeostasis for mitochondrial activity is well recognized, direct evidence linking cytosolic acidification to mitochondrial dysfunction in a physiological context has been lacking. Whilst the mode of action is unclear at this point, increased H_2_O_2_ has been shown to result in cytochrome *c* release (Elena-Real et al. 2021). Notably, a small release of cytochrome *c* in the cytosol was detected within 10 min after SI induction in *Papaver* pollen tubes, preceding the more pronounced increase observed after 1 h (Thomas and Franklin-Tong 2004). This early release could contribute to a decrease in electron transport rate. Overall, we hypothesize that SI triggers disruption of ΔΨ_m_, most likely *via* inhibition of mitochondrial electron transport, alongside oxidative inhibition of glycolysis and the TCA cycle. The resulting drop in ATP may, in turn, potentiate cytosolic acidification, at least in part *via* decreased plasma membrane and/or vacuolar H^+^-ATPase activity. Indeed, inhibition of mitochondrial electron transport results in cytosolic acidification, which has been attributed to impaired plasma membrane H^+^-ATPase activity due to low ATP supply (Khan et al. 2024). However, this alone is unlikely to account for the acidification observed following SI, suggesting that additional mechanisms must be involved (Wang et al. 2022). Recent characterization of the pollen mitochondrial proteome (Boussardon et al. 2025) has revealed unique features associated with high respiration rates needed for rapid pollen tube tip growth, including high abundance of TCA cycle enzymes, electron transport complexes, and Ca^2+^ homeostasis and a deficiency of transcriptional and translational machinery. These specialized features may render pollen highly susceptible to the kind of disruption observed during SI. Collectively, these findings support a model in which mitochondrial dysfunction and metabolic inhibition form a self-reinforcing feedback loop, amplifying cellular disruption (Fig. 10).

### Linking early mitochondrial disruption to PCD

We have shown that mitochondria, as well as acting as the major source of SI-induced ROS, also undergo collapse of Δψ_m_. This provides direct evidence of compromised mitochondrial function, complementing previous observations in *Papaver* pollen tubes of cytochrome *c* release (Thomas and Franklin-Tong 2004) and altered mitochondrial morphology (Geitmann et al. 2004), both hallmarks of reduced mitochondrial quality and PCD. Our findings position Δψ_m_ collapse within a broader framework of SI-induced mitochondrial dysfunction, where mitochondria serve as both sources and sensors of redox imbalance upstream of PCD. This dual role is likely to accelerate the coupling of ROS signals to metabolic shutdown and PCD commitment. SI triggers PCD downstream of early signals (Thomas and Franklin-Tong 2004; Wang et al. 2019; Bosch and Franklin-Tong 2024). Although the current study did not directly examine PCD, several of the rapid events triggered by SI, such as mitochondrial dysfunction, ROS production, and ATP depletion, have previously been associated with PCD in plants (Xie et al. 2014; Van Aken and Van Breusegem 2015; Huang et al. 2016). While the role of mitochondria in plant PCD remains rather contentious and an area of ongoing debate, it has been suggested that they can act as early integrators of PCD signaling in certain systems (Curtis and Wolpert 2004; Mur et al. 2008; Møller et al. 2021). Our previous studies on *Papaver* have shown that SI induces the release of cytochrome *c* within 10 min, followed by morphological changes, including swelling and loss of cristae (Geitmann et al. 2004; Thomas and Franklin-Tong 2004 ). These alterations resemble mitochondrial changes associated with apoptosis in animal cells, and similar mitochondrial morphology transitions have also been linked to PCD in plants (Scott and Logan 2008; Ocampo-Hernández et al. 2022). Combined with our findings of ATP depletion and mitochondrial dysfunction, this demonstrates that mitochondria are early targets of SI-induced signaling to PCD.

Studies to date suggest that many plant PCD processes, such as those in the stigma, tapetum, or hypersensitive response, involve vacuolar rupture and NADPH oxidase-derived ROS (Franklin-Tong and Bosch 2021; Zhang et al. 2021; Bosch and Franklin-Tong 2024; Kacprzyk et al. 2024). However, our findings here demonstrate that mitochondrially-derived ROS contribute to metabolic dysfunction that underpins PCD in the SI system. This, together with previous data, clearly implicates very early mitochondrial alterations observed within minutes (Thomas and Franklin-Tong 2004) as being involved as a causal event upstream of PCD (and not just a marker of dying cells). Consistent with this, recent work in Arabidopsis root cap cells showed that mitochondrial disruption, including the release of matrix proteins, is one of the earliest detectable events during developmental cell death, preceding the breakdown of other organelles (Wang et al. 2024). Thus, there are clearly distinct signals and outputs that are responsible for PCD, underlining the diversity of plant PCD systems. Establishing how rapid changes in intracellular Ca^2+^, pH, and redox state influence the downstream commitment to PCD remains a key challenge for the field.

## Conclusions

We have provided an integrated analysis of the complex cascade of cellular and metabolic events that involve potential positive feedback loops triggered during the early stages of the SI response (Fig. 10), using a combination of cell imaging and metabolic assays. Focusing on the first 20 min of this response, we show that a Ca^2+^-and pH-dependent series of events results in mitochondrial ROS production, likely *via* disruption of mitochondrial electron transport. This results in inhibition of mitochondrial respiration and ATP production. Concurrently, oxidative signals are observed in the cytosol, oxidizing and inhibiting the glycolytic enzyme GAPDH, further impacting ATP production. Our data implicate the mitochondria as a central hub of SI-triggered metabolic collapse and ROS signaling that is triggered by increases in [Ca^2+^]_cyt_ and [pH]_cyt_. However, the precise molecular mechanisms by which the rapid Ca^2+^ and pH changes perturb mitochondrial function and ROS production remain to be elucidated, representing an important area for future investigation.

## Methods

### Plant material and growth conditions

Transgenic *Arabidopsis thaliana* lines expressing PrpS_1_ in pollen, along with various fluorescent markers (see Table S1), were used in this study. Plants were grown in controlled environment chambers under a 16-h light/8-h dark photoperiod, with a light intensity of 135 μmol m⁻^2^ s⁻^1^. Mature pollen grains were harvested from fully opened flowers. *Papaver rhoeas* pollen grains were collected from field-grown plants and stored over silica gel at −20 °C before use, as described previously (Snowman et al. 2002).

### Plasmid construction and generation of transgenic plants

Details of primers, targeting sequences for roGFP2-Orp1, and Golden Gate modules used are listed in Table S2.

The dual-expression vector carrying *ProNTP303:PrpS_1_-ProNTP303:LifeAct-mRuby2* was generated using GreenGate cloning (Lampropoulos et al. 2013). Promoter *NTP303* was amplified using primer sets *F-A-NTP303/R-B-NTP303*, *F-D-NTP303/R-E-NTP303* with the vector carrying *ProNTP303:PrpS_1_-GFP* as the template (de Graaf et al. 2012). The PCR products were cloned into pJET1.2 using CloneJET PCR Cloning Kit (ThermoFisher) to obtain the entry vectors *pEN-A-ProNTP303-B* and *pEN-D-ProNTP303-E*. Generation of other entry vectors, including *pEN-B-PrpS1-C*, *pEN-C-tRBCS-D*, *pEN-E-LifeAct-mRuby2-F,* and *pEN-F-tMAS-G,* was described in Lin et al. (2020). These entry clones were assembled into GreenGate destination vector *pFAST-GK-AG* to produce the dual-expression vector carrying *ProNTP303:PrpS_1_-ProNTP303:LifeAct-mRuby2*.

Constructs for roGFP2-Orp1 localization in the cytosol, mitochondria, plastids, peroxisomes, and nuclei were assembled using Golden Gate reactions (Weber et al. 2011; Engler et al. 2014), following the procedures described in Arnaud et al. (2023) with the following modifications.

To ensure pollen-specific expression, the CaMV 35S promoter (*Pro35S*) was replaced with the pollen-specific promoter *ProNTP303* (Weterings et al. 1995). *ProNTP303* was amplified using primer sets NTP303_F/NTP303_AATG_R and NTP303_F/NTP303_TAACT_R as described above. These amplified fragments were cloned into pICH41233 and pICH41295, respectively. The pICH41295-ProNTP303 module was used for assembling constructs containing a nuclear localization signal (NLS), whereas the pICH41233-ProNTP303 module was used for assembling constructs containing a mitochondria-targeting signal (MTS), chloroplast (Chl)- and peroxisome-targeting signal serine-lysine-leucine (SKL).

The introduction of targeting sequences for the localization of roGFP2-Orp1 to subcellular compartments followed procedures as described in Arnaud et al. (2023). For the MTS, a sequence encoding the first 54 amino acids of the transit peptide of the Arabidopsis homologue (At5g08680) of the *Nicotiana plumbaginifolia* ATP synthase beta-3 subunit (with 98% sequence identity, Chaumont et al. 1994; Carrie et al. 2015) was amplified using Arabidopsis Col-0 cDNA as the template with primers MTS­_F and MTS­_R. The chloroplast-targeting signal was amplified from pICH78133 (Engler et al. 2014) using primers Chloro_F and Chloro_R. These fragments were subsequently cloned into pICH41246.

The plasmid carrying *ProNTP303:Lifeact-mRuby2-ProNTP303:PrpS_1_* was transformed into *Arabidopsis thaliana* Col-0. Constructs for roGFP2-Orp1 localization were transformed into Arabidopsis plants carrying *ProNTP303:Lifeact-mRuby2-ProNTP303:PrpS_1_*. *Agrobacterium tumefaciens* GV3101 and the floral dip method (Clough and Bent 1998) were used for transformation. Transgenic seedlings were selected and screened based on fluorescence intensity for strong expression, which were used for further analyses.

### Pollen tube growth and treatment

*Arabidopsis thaliana* and *Papaver rhoeas* pollen were germinated *in vitro* in liquid growth medium for over 60 min before treatment and imaging, as previously described (Snowman et al. 2002; Wang et al. 2020). Self-incompatibility responses were induced by adding recombinant PrsS_1_ to *Arabidopsis* pollen tubes expressing PrpS_1_ or PrsS_1_ and PrsS_3_ to *Papaver* pollen tubes (*S*_1_*S*_3_) to a final concentration of 20 μg ml^−1^ as described previously (Wilkins et al. 2015; Wang et al. 2020). H_2_O_2_ (Sigma-Aldrich) or dithiothreitol (DTT, Thermo Fisher Scientific) was applied at concentrations indicated in the results. To evaluate the effect of SI on the reductive capacity of roGFP2-Orp1, SI was induced for 1.5 min, followed by a 5-min treatment with 2.5 mM H_2_O_2_ before imaging. To evaluate mitochondrial membrane potential, *Arabidopsis* pollen tubes were treated with 1 μM tetramethylrhodamine methyl ester (TMRM, Thermo Fisher Scientific) and washed with growth medium before imaging (Scaduto and Grotyohann 1999; Perry et al. 2011). Various compounds (Sigma-Aldrich), including Diphenyleneiodonium chloride (DPI), antimycin A (AA), carbonyl cyanide-p-trifluoromethoxyphenylhydrazone (FCCP), and calcium Ionophore A23187, were applied at concentrations detailed in the results section. The carrying solvents, DMSO (except ethanol for AA), at a final concentration of no greater than 0.1% (v/v) in growth medium, had no effect on pollen tube growth. GdCl_3_ and caffeine were applied at 500 μM and 10 mM, respectively. To preserve the redox state of roGFP2-Orp1 after PrsS or drug treatment, N-ethylmaleimide (NEM, Sigma-Aldrich) was used at 20 mM (Wendt et al. 2022). Cytosolic pH was altered using 50 mM propionic acid at pH 7.0 or 5.5, as previously described (Wilkins et al. 2015).

### Visualization of tip-localized superoxide production

Superoxide production at the pollen tube tip was detected by 5 mg ml^−1^ nitroblue tetrazolium (NBT, Sigma-Aldrich) reduction for 15 min. Images were captured using a Leica DMi8 microscope (100× CS2 objective, NA 1.40) equipped with a Leica TCS SPE camera. Grey values within the cytosolic region 0 to 50 μm from the apex were quantified using Fiji software (Schindelin et al. 2012).

### Confocal imaging and image analyses

Fluorescence markers were imaged using a Leica SP8 confocal microscope (×100 CS2 objective, NA 1.40). Lifeact-mRuby2 decorating F-actin, and sensors for [Ca^2+^]_cyt_ (Yellow Cameleon 3.6, YC3.6) and [pH]_cyt_ (pHGFP) were imaged as previously described (Wang et al. 2020). roGFP2-Orp1 was sequentially excited at 405 and 488 nm, with emission collected at 505 to 535 nm. For roGFP2-Orp1 targeting the cytosol, the pinhole was set to 5 airy units (Nietzel et al. 2019), and for subcellular compartments, it was set to 1 airy unit. TMRM was excited at 561 nm, and emission was collected at 650 to 775 nm. For each pollen tube, 11 z-stack images were taken with 0.9 μm intervals. Fluorescent intensity ratios (R_405/488_) for roGFP2-Orp1 were processed and quantified using Fiji software (Schindelin et al. 2012). The 0 to 40 μm from the pollen tube tip was selected as the region of interest (ROI). Background noise was subtracted using “Subtract background” with a radius of 50 pixels. Ratios for YC3.6 (R_Venus/CFP_) and pHGFP (R_405/488_) were processed and quantified using Leica Application Suite X (LAS X). Additional image processing for figure preparation was conducted using Fiji software (Schindelin et al. 2012). Measurements were performed on individual pollen tubes, with three independent experiments conducted per treatment.

### ATP assays

Intracellular ATP levels in *Arabidopsis* pollen tubes were quantified using the Luminescent ATP Detection Assay Kit (Abcam), as previously described (Wang et al. 2022).

### Measurement of pollen tube oxygen consumption

REDFLASH fiber optic oxygen sensors (FireSting-O_2_; PyroScience; Aachen, Germany) were used to measure oxygen uptake (Bénit et al. 2017). After being hydrated in a humid chamber for 50 min, *Papaver* pollen was grown in 2 ml glass vials partially filled with wax and then completely filled with a known volume (∼0.4 ml) of liquid *Papaver* GM. The vials were sealed with caps that allowed insertion of the probe and the addition of treatments, including 2.5 to 10 mM H_2_O_2_ and the mixture of PrsS_1_ and PrsS_3_ or PrsS_3_ and PrsS_8_ (50 to 100 mg ml^−1^). Oxygen concentration was measured in the liquid phase using the Pyro Oxygen Logger software. Respiration rate was determined by calculating the rate of oxygen uptake over defined time windows. For the data shown in Fig. 8, each rate represents the average oxygen uptake over a 30 min interval, normalized to the weight of pollen. As it takes 30 min to obtain a datapoint for oxygen uptake, the 30 to 60 min and 60 to 90 min data were normalized against the mean uptake rate during the initial 0 to 30 min period.

### GAPDH activity assays

Glyceraldehyde 3-phosphate dehydrogenase (GAPDH) activity in pollen extracts was assessed by measuring NADH formation in the presence of glyceraldehyde 3-phosphate and arsenate. *Papaver* pollen was grown on solidified *Papaver* GM for 2 h before the addition of treatments, including 2.5 mM H_2_O_2_ and a mixture of recombinant PrsS_1_ and PrsS_3_ proteins (50 to 100 mg ml^−1^) for 5 to 30 min. Arabidopsis pollen was grown on solidified Arabidopsis GM for 4 to 5 h before the addition of treatments including 2.5 mM H_2_O_2_ and recombinant PrsS_1_ or PrsS_3_ proteins (50 to 100 mg ml^−1^) for 5 min. Pollen tube samples were collected in liquid nitrogen and ground into enzyme extraction buffer containing 50 mM HEPES/KOH pH 7.5, 10% (v/v) glycerol, 0.25% (w/v) BSA, 0.1% (v/v) Triton X-100, 10 mM MgCl_2_, 1 mM EDTA, 1 mM EGTA, 1 × protease inhibitor cocktail, 1 mM PMSF and 1 mM DTT (Gibon et al. 2004). After centrifugation, enzyme extraction in the supernatant was quantified by the standard Bradford assay (Bio-Rad). Glycolytic GAPDH activity was monitored spectrophotometrically at 340 nm *via* the production of NADH by using a ClarioStar plate reader (BMG LABTECH, Aylesbury, UK). The oxidation of glyceraldehyde-3-phosphate to 1,3-bisphosphoglycerate was measured in an assay buffer containing 20 mM Tris-HCl, pH 7.5, 1.25 mM EDTA, 15 mM sodium arsenate, and 1.25 mM NAD as described in Hillion et al. (2017). After mixing the enzyme extraction with the assay buffer, the reaction was started by the addition of 0.25 mM glyceraldehyde-3-phosphate in a 96-well UVStar microplate (Greiner Bio-One). Sodium arsenate was used as a co-substrate to form unstable 1-arseno,3-phosphoglycerate, whose degradation allows a favorable equilibrium for measuring the rate of GAPDH activity in the glycolytic forward reaction. The GAPDH specific activity was calculated from the increase in absorbance per minute using the extinction coefficient of NADH (6.22 mM^−1^ cm^−1^) and protein concentration.

### Pollen metabolites

*Arabidopsis* pollen tubes were germinated in growth medium for 2 h before treatment. Pollen grains from 75 flowers were used per sample. Numbers of pollen tubes was estimated by measuring the grey values within images of the pollen tube growth field. Pollen tubes were collected by centrifugation at 6,000 × *g* for 5 min at 22 °C before flash freezing using liquid nitrogen. Organic acids and glutathione were measured by LC-triple quadrupole MS/MS following derivatization of pollen extracts with O-benzylhydroxylamine (Walvekar et al. 2018). 20 µl methanol/acetonitrile/0.1 M formic acid in water (4:4:2) was added to the pelleted pollen in 2 ml plastic centrifuge tubes, followed by freeze-thawing (−70 °C) and sonication for 20 min in an ice-cold water bath. Derivatization reagent (100 µl) was added, and the samples incubated for 60 min. 20 µl of 0 to 20 µM of a standard mixture and blanks without pollen were also derivatized. The derivatization reagent was prepared by mixing equal volumes of 1 M O-benzylhydroxylamine hydrochloride dissolved in acetonitrile/water (3:2) and 1 M N-(3-dimethylaminopropyl)-N′-ethylcarbodiimide dissolved in water/pyridine/37% HCl (16:1.6:1, pH 5.0). Ethyl acetate (300 µl) was added to each sample, followed by vortexing and centrifugation (1,400 × *g*, 5 min, 4 °C). The upper ethyl acetate phase containing the O-benzylhydroxylamine derivatives was transferred to tapered glass autosampler vials and dried down under vacuum, followed by dissolving in 20 µl 50% methanol. The samples were analysed with an Agilent 6420B triple quadrupole mass spectrometer coupled to a 1200 series Rapid Resolution HPLC system (Agilent Technologies, Santa Clara, USA). Samples and standards (5 µl) were injected onto a Polaris 3 C18-A (4.6 × 100 mm, 3.0 µm particle size) column with a Polaris 5 C18-A (10 × 2 mm) guard column (Agilent Technologies, Santa Clara, USA) at 25 °C and eluted at 0.3 ml min^−1^ with solvent A (0.1% (v/v) formic acid and 10% (v/v) methanol in water) and solvent B (0.1% (v/v) formic acid in methanol). The elution gradient was 10% B from 0 to 0.5 min, 95% B at 11 min, 95% B from 11 to 15 min, and 10% B at 26 min. The triple quadrupole source conditions were as follows: gas temperature 350 °C, drying gas flow rate 9 l min^−1^, nebulizer pressure 35 psig, and capillary voltage 4 kV. Compounds were detected by multiple reaction monitoring in positive ion mode, and the fragmentor voltage, collision energies, along with precursor and product ion m/z for each compound are shown in Table S3. Peak areas for each compound were extracted with MassHunter Quantitative Analysis software (v10.2, Agilent Technologies, Santa Clara, USA) and normalized to pollen number.

### Accession numbers

*Papaver rhoeas* PrpS1 (CAN86556.1) and PrsS (Q40975.1) are available in GenBank.

## Supplementary Material

koag031_Supplementary_Data

## Data Availability

The data underlying this article are available in the article and in the online supplementary material.

## Dive Curated Terms

The following phenotypic, genotypic, and functional terms are of significance to the work described in this paper:

NADPH CHEBI: CHEBI:16474

pollen AmiGo: PO:0025281

NADH CHEBI: CHEBI:16908

FCCP CHEBI: CHEBI:75458
