# Supplementary material for: Papaver S-determinants trigger mitochondrially derived ROS production and disrupt energy metabolism in incompatible pollen tubes
Source: Plant Cell. 2026 Feb 19;38(3):koag031. doi: 10.1093/plcell/koag031 (PMC13098185; doi:10.1093/plcell/koag031)
Supplement: koag031_Supplementary_Data [file koag031_Supplementary_Data.zip › Supplementary Figures and Tables S1-S3.pdf]

Papaver S-determinants trigger an integrated network of mitochondrially derived ROS and disruption of energy metabolism in incompatible pollen tubes

Ludi Wang[1,*], An-Shan Hsiao[2,], José Carli[1], Ali Raza[1], Zongcheng Lin[3], Dominique Arnaud[2], Julia M. Davies[4], Vernonica E. Franklin-Tong[5], Nicholas Smirnoff[2] , Maurice Bosch[1]**

[1]Institute of Biological, Environmental and Rural Sciences (IBERS), Aberystwyth University, Plas Gogerddan, Aberystwyth, SY23 3EE, UK

[2] Biosciences, Faculty of Health and Life Sciences, University of Exeter, Exeter, EX4 4QD, UK

[3]National Key Laboratory for Germplasm Innovation & Utilization of Horticultural Crops, College of Horticulture and Forestry Sciences, Huazhong Agricultural University, Wuhan 430070, China; Hubei Hongshan Laboratory, Wuhan 430070, China.

[4]Department of Plant Sciences, University of Cambridge, Downing Street, Cambridge, CB2 3EA, UK

[5]School of Biosciences, College of Life and Environmental Sciences, University of Birmingham, Edgbaston, Birmingham, B15 2TT, UK

[*]Current address: Sainsbury Laboratory University of Cambridge, Cambridge, CB2 1LR, UK; ** Current address: School of Biosciences, College of Life and Environmental Sciences, University of Birmingham, Edgbaston, Birmingham, B15 2TT, UK

Correspondence: Maurice Bosch (mub@aber.ac.uk), Ludi Wang (lw821@cam.ac.uk), Vernonica Franklin-Tong (v.e.franklin-tong@bham.ac.uk) and Nicholas Smirnoff (N.Smirnoff@exeter.ac.uk)

Figure S1. Disruption of tip-localized NADPH oxidase activity in *Papaver* pollen tubes following SI induction.

Figure S2. *In vivo* characterisation of roGFP2-Orp1 targeted to various subcellular compartments in Arabidopsis pollen tubes.

Figure S3. Imaging of mitochondria- and plastid-targeted roGFP2-Orp1 in Arabidopsis pollen tubes before and after SI induction.

Figure S4. SI does not impair the reduction capacity of roGFP2-Orp1 in the cytosol and mitochondria of Arabidopsis pollen tubes.

Figure S5. Antimycin A induces rapid decrease in mitochondrial membrane potential in Arabidopsis pollen tubes.

Figure S6. Manipulation of $[Ca^{2+}]_{cyt}$ in Arabidopsis pollen tubes by A23187 and $GdCl_3$.

Figure S7. GAPDH activities in *Papaver* pollen tubes after SI induction and H_2O_2 treatment.

Figure S8. Changes in TCA cycle metabolite levels in Arabidopsis pollen tubes following SI induction.

Table S1. Transgenic Arabidopsis lines used in this study.

Table S2. Oligonucleotides, targeting peptide sequences and Golden Gate modules used for transgene construction.

Table S3. Multiple reaction monitoring (MRM) transitions for LC-triple quadrupole MS/MS analysis of TCA cycle acids and glutathione.

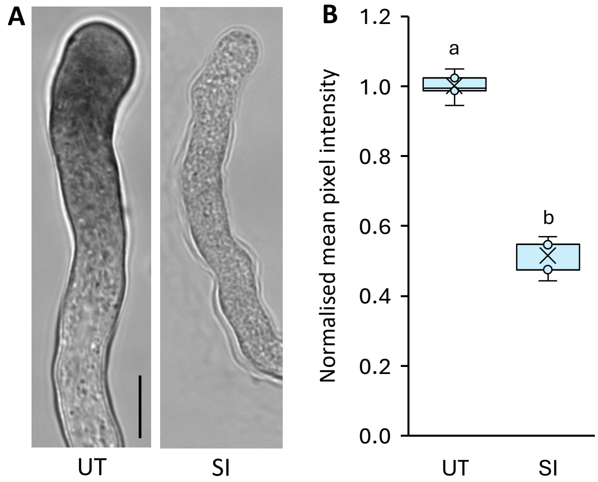

Figure S1. Disruption of tip-localized NADPH oxidase activity in *Papaver* pollen tubes following SI induction. (A) Nitroblue tetrazolium (NBT) staining was used to detect superoxide production at the tips of *Papaver* pollen tubes during the SI response. Representative images show an untreated (UT) pollen tube and a pollen tube treated with $PrsS_1$ (SI) for 15 min. NBT staining was significantly reduced after SI induction. Scale bar = 10 µm. (B) Quantification of NBT staining intensity at pollen tube tips. Reduced NBT staining reflects decreased pixel intensity, indicating a reduction in superoxide production. Different letters indicate significant differences ($P < 0.001$; Tukey's test). n = 5.

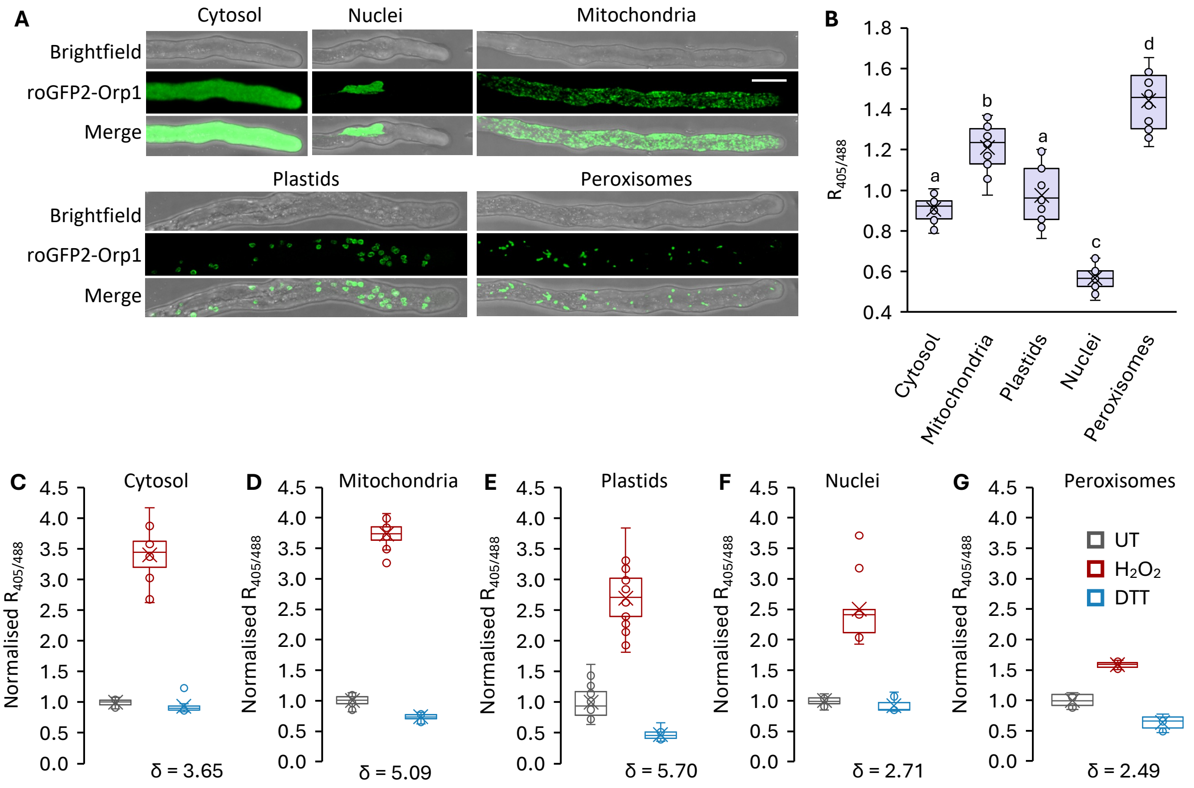

Figure S2. *In vivo* characterisation of roGFP2-Orp1 targeted to various subcellular compartments in Arabidopsis pollen tubes. (A) Subcellular localisation of roGFP2-Orp1 targeted to the cytosol, mitochondria, plastids, peroxisomes and nuclei. Representative images show fluorescence emission detected at 505–535 nm after excitation at 488 nm. Scale bar = 10 µm. (B) Initial oxidation states of roGFP2-Orp1 in the cytosol, mitochondria, plastids, peroxisomes and nuclei. Different letters indicate significant differences ($P < 0.001$) based on Tukey's test n ≥ 17. (C-G) Dynamic range characterisation of roGFP2-Orp1 oxidation and reduction in response to H_2O_2 and DTT in various subcellular compartments. Pollen tubes expressing roGFP2-Orp1 targeted to the cytosol (C), mitochondria (D), plastids (E), nuclei (F) and peroxisomes (G) were treated with 15 mM H_2O_2 or 10 mM DTT. The 405/488 nm excitation fluorescence ratio ($R_{405/488}$) was measured via live-cell imaging and normalised to the initial mean $R_{405/488}$. The dynamic range (δ) of roGFP2-Orp1 in each compartment was calculated using the $R_{405/488}$ after oxidation (H_2O_2 treatment) and reduction (DTT treatment). n ≥ 10.

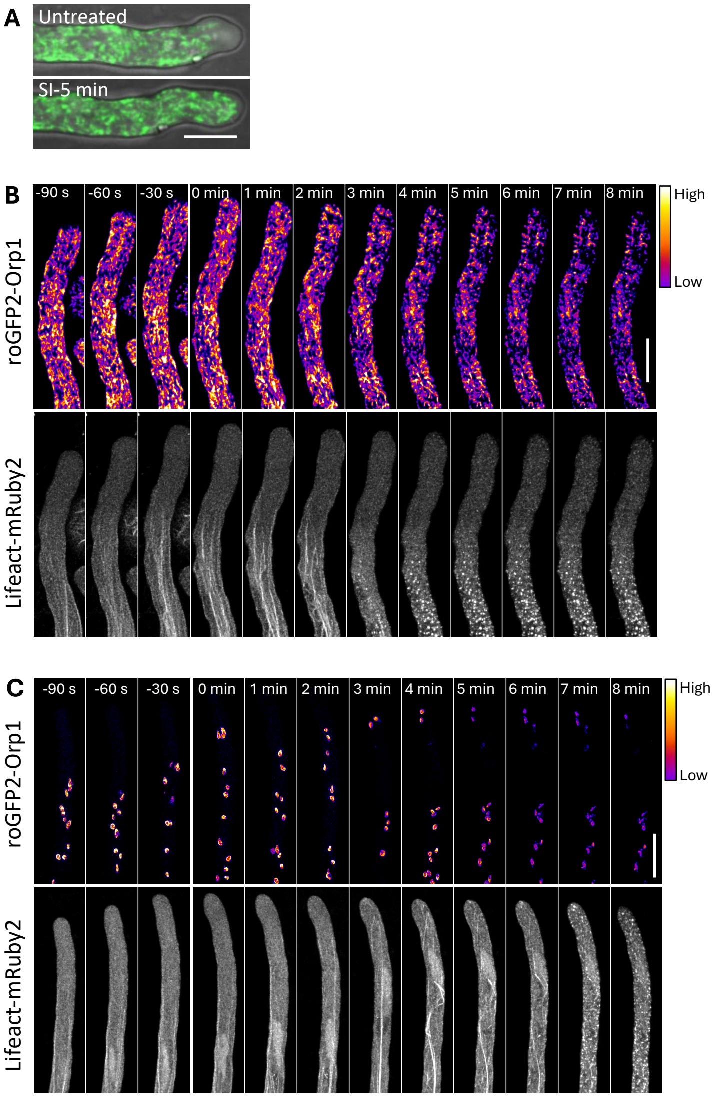

Figure S3. Imaging of mitochondria- and plastid-targeted roGFP2-Orp1 in Arabidopsis pollen tubes before and after SI induction. (A) Representative images showing the distribution of mitochondria-targeted roGFP2-Orp1 fluorescence excited at 488 nm and detected at 505-535 nm before (untreated) and after 5 minutes of SI induction. (B-C) Representative time-lapse images showing roGFP2-Orp1 fluorescence in mitochondria (B) or plastids (C) of Arabidopsis pollen tubes, excited at 488 nm and detected at 505-535 nm (upper panel) before and after SI induction. The lower panel shows F-actin labelled with lifeact-mRuby2. The colour gradient indicates the fluorescence intensity, which decreases after SI induction due to oxidation of the sensor. Scale bar = 10 μm.

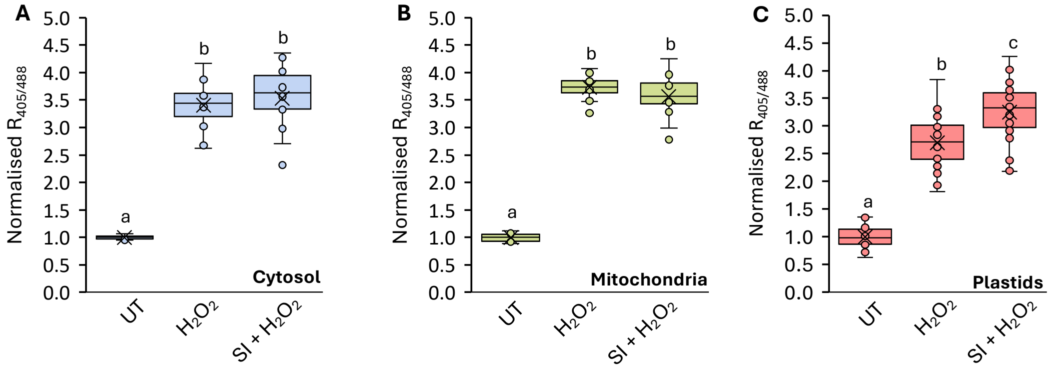

Figure S4. SI does not impair the reduction capacity of roGFP2-Orp1 in the cytosol and mitochondria of Arabidopsis pollen tubes. Quantification of 405/488 nm fluorescence ratio ($R_{405/488}$) of roGFP2-Orp1 targeted to the cytosol (A), mitochondria (B) and plastids (C) in untreated (UT) pollen tubes or after 5 minutes of H_2O_2 treatment, with and without a preceding 1.5-minute SI induction. Sample sizes: A, n ≥ 10. B, n = 15. C, n ≥ 10. Different letters indicate significant differences based on Tukey's test ($P < 0.001$).

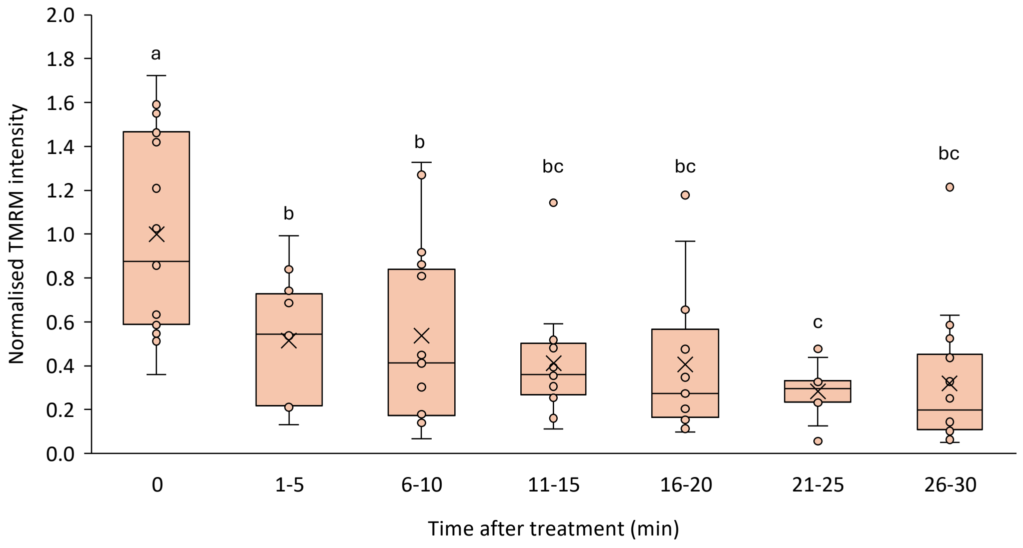

Figure S5. Antimycin A induces rapid decrease in mitochondrial membrane potential in Arabidopsis pollen tubes. Quantification of TMRM fluorescence in mitochondria after treatment with antimycin A (AA). Different letters indicate significant ($P < 0.05$) differences based on Tukey's test (compact letter display). n = 24, 10, 15, 16, 15, 10, 18.

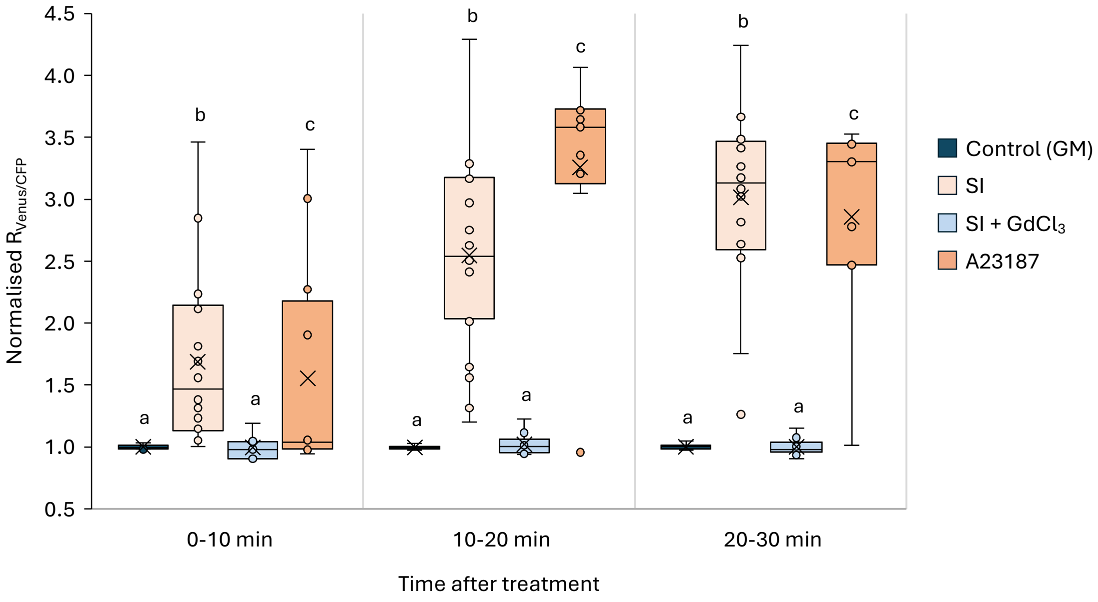

Figure S6. Manipulation of $[Ca^{2+}]_{cyt}$ in Arabidopsis pollen tubes by A23187 and $GdCl_3$. Quantification of YC3.6 fluorescence ratio ($R_{Venus/CFP}$) in pollen tubes treated with growth medium (GM), $PrsS_1$ (SI), $PrsS_1$ in combination with $GdCl_3$ (SI + $GdCl_3$), or calcium ionophore (A23187). $n \geq 9$. $R_{Venus/CFP}$ values were normalised to the mean of GM-treated samples within the first 10 minutes of treatment. Different letters indicate significant differences based on Tukey's test ($P < 0.001$).

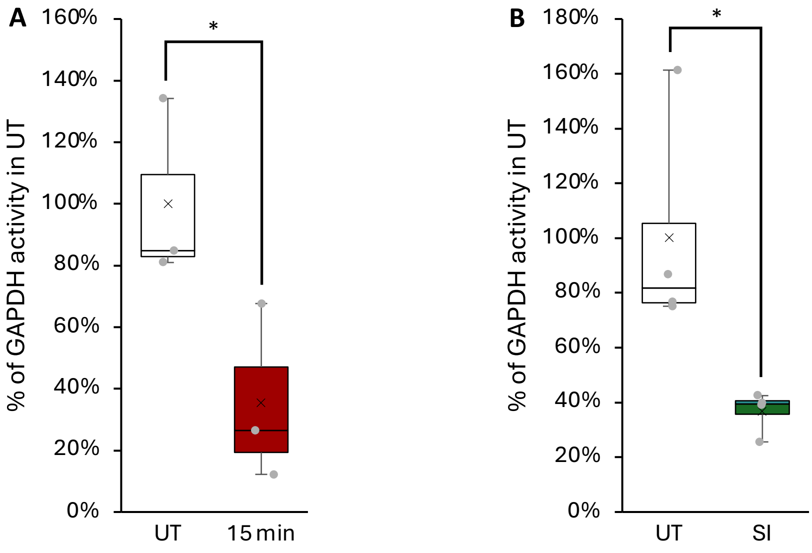

Figure S7. GAPDH activities in *Papaver* pollen tubes after SI induction and H_2O_2 treatment. GAPDH activities were measured in *Papaver* pollen tubes 15 minutes after treatment with 2.5 mM H_2O_2 (**P* < 0.05, Mann-Whitney U test, n = 3) and 30 minutes after SI induction (**P* < 0.05, Mann-Whitney U test, n = 4). All GAPDH activities were normalised to the mean values of untreated (UT) or control samples.

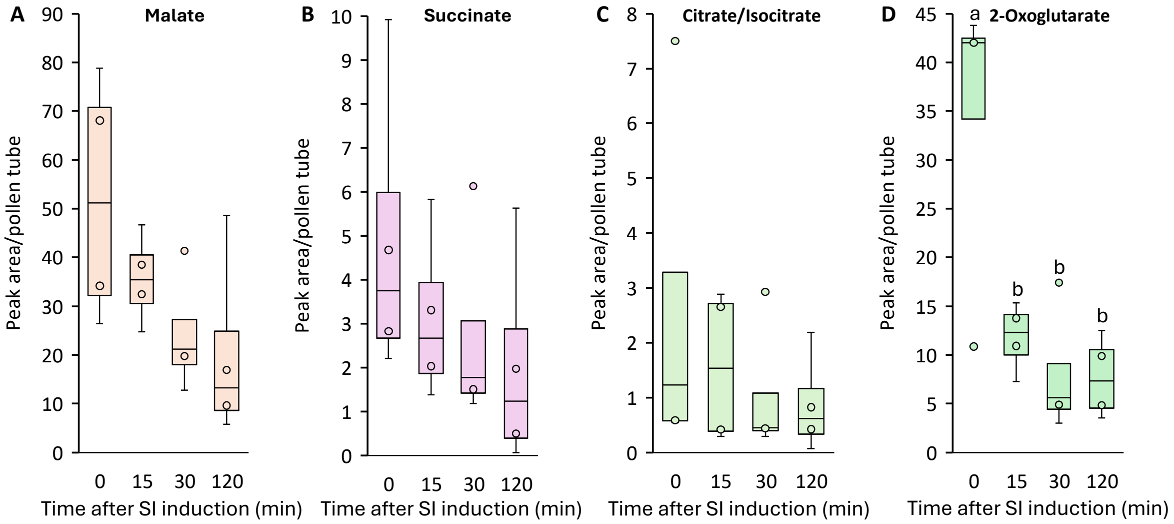

Figure S8. Changes in TCA cycle metabolite levels in Arabidopsis pollen tubes following SI induction. Metabolite levels of malate (A), succinate (B) and citrate/isocitrate (C) decreased over the 0-120 minute period following SI induction, though not significantly. In contrast, 2-oxoglutarate (D) showed a significant decrease starting 15 minutes after SI induction. Different letters indicate significant differences based on Tukey's test ($P < 0.001$, n = 4).

Table S1. Transgenic Arabidopsis lines used in this study.

Lines	Constructs	Corresponding figures
1	*ProNTP303:roGFP2-Orp1, ProNTP303:Lifeact-mRuby2-ProNTP303:PrpS$_1$*	Figure 1, Figure S2, Figure 2A and 2F, Figure 3, Figure S4A, Figure 4A and 4C, Figure 6F and 6G, Figure 7A
2	*ProNTP303:Mito-roGFP2-Orp1, ProNTP303:Lifeact-mRuby2-ProNTP303:PrpS$_1$*	Figure S2, Figure 2B and 2G, Figure S3A, Figure S4B, Figure 4B, Figure 6D, Figure 7B
3	*ProNTP303:Chl-roGFP2-Orp1, ProNTP303:Lifeact-mRuby2-ProNTP303:PrpS$_1$*	Figure 2C and 2H, Figure S3B, Figure S4C, Figure 4D, Figure 6E, Figure 8C-E, Figure 9, Figure S8
4	*ProNTP303:roGFP2-Orp1-SKL, ProNTP303:Lifeact-mRuby2-ProNTP303:PrpS$_1$*	Figure S2, Figure 2D
5	*ProNTP303:NLS-roGFP2-Orp1, ProNTP303:Lifeact-mRuby2-ProNTP303:PrpS$_1$*	Figure S2, Figure 2E
6	*ProNTP303:pHGFP-ProNTP303:PrpS$_1$ (Wang et al., 2020)*	Figure 5, Figure 6B, Figure 7C-D, Figure S5
7	*ProNTP303:YC3.6-ProNTP303:PrpS$_1$ (Wang et al., 2020)*	Figure 6A and 6C, Figure S6,

Table S2. Oligonucleotides, targeting peptide sequences and Golden Gate modules used for transgene construction. A. List of oligonucleotides used in this study. F, forward primers. R, reverse primers. Bold letters indicate target-binding sequences. B. Targeting peptide sequences fused to roGFP2-Orp1 for subcellular localisation; C. Golden Gate Assembly modules used in this study.

A

Primer name	Sequence (5'-3')
F-A-NTP303	*TTTTGGTCTCAACCT**GATACACTCGCAACGTGTG**TATCCTAA*
R-B-NTP303	*TTTTGGTCTCATGTT**GACGTTGTTTTTTTTATT**CTTTAATCCCC*
F-D-NTP303	*TTTTGGTCTCATCAG**GATACACTCGCAACGTGTG**TATCCTAA*
R-E-NTP303	*TTTTGGTCTCAGCAG**GACGTTGTTTTTTTTATT**CTTTAATCCCC*
NTP303_F	*TTGAAGACAA**GGAGATACACTCGCAACGTGTG***
NTP303_TACT_R	*TTGAAGACAA**AGTAGACGTTGTTTTTTTTATT***
NTP303_AATG_R	*TTGAAGACAA**CATTGACGTTGTTTTTTTTATT***
Chloro_F	*TTGAAGACAA**ATACTATGGCTTCTTCTATGCTTTCTTCTGC***
Chloro_R	*TTGAAGACAA**CATTGCTCGAACTCTTCCTCCGTTACTAG***
NLS_F	*TTGAAGACAA**TACTATGCTGCAGCCTAAGAAGAAGAGAAAGGTTGGAGGAATGTTGTCTTCAA***
NLS_R	*TTGAAGACAA**CATTCCTCCAACCTTTCTCTTCTTCTTAGGCTGCAGCATAGTATTGTCTTCAA***
MTS_F	*TTGAAGACAAA**TACTATGGCGAGTCGGCGAATCTT***
MTS_R	*TTGAAGACAA**CATTTTTGCCACCGTAATCATAGG***

B

Targeting compartments	Targeting sequences	References
Cytoplasm	Nuclear Export Signal (NES) of the protein kinase inhibitor (PKI) from rabbit	Wen et al., 1995; Arnaud et al., 2023
Mitochondria	Mitochondrial targeting signal of the ATPase β subunit from *Arabidopsis thaliana* (98% sequence identity with *Nicotiana plumbaginifolia*)	Sjöling & Glaser, 1998, Carrie et al., 2015
Chloroplasts	Chloroplast transit peptide from the Rubisco small subunit RbcS (synthetic consensus of dicot sequences)	Marillonnet et al., 2004; Engler et al., 2014; Arnaud et al., 2023
Peroxisomes	SKL sequence for targeting to peroxisome	Sparkes et al., 2003; Arnaud et al., 2023
Nuclei	Nuclear localisation signal derived from Simian Virus 40 (NLS SV40)	Kalderon et al., 1984; Arnaud et al., 2023

C

Modules used	GoldenGate assembly level	*Modules/constructs produced*	*Reference*
pICH41233, PCR product ProNTP303	*0*	*pICH41233-ProNTP303*	*Engler et al., 2014; de Graaf et al., 2012*
pICH41295, PCR product ProNTP303	*0*	*pICH41295-ProNTP303*	*Engler et al., 2014; de Graaf et al., 2012*
pICH41246, PCR product MTS_F+R	*0*	*pICH41246-MTS*	*Engler et al., 2014*
pICH41246, PCR product Chloro_F+R	*0*	*pICH41246-Chl*	*Engler et al., 2014*
pICH47732, pICH41233-ProNTP303, pICH41308-roGFP2-Orp1, pICH41421	*1*	*ProNTP303:roGFP2-Orp1-NOS*	*Arnaud et al., 2023*
pICH47732, pICH41233-ProNTP303, pICH41246-MTS, pICH41308-roGFP2-Orp1, pICH41421	*1*	*ProNTP303:MTS-roGFP2-Orp1-NOS*	*Arnaud et al., 2023*
pICH47732, pICH41233-ProNTP303, pICH41246-Chl, pICH41308-roGFP2-Orp1, pICH41421	*1*	*ProNTP303:Chl-roGFP2-Orp1-NOS*	*Arnaud et al., 2023*
pICH47732, pICH41295-ProNTP303, pICH41308-roGFP2-Orp1-SKL, pICH41421	*1*	*ProNTP303:roGFP2-Orp1-SKL-NOS*	*Arnaud et al., 2023*
pICH47732, pICH41295-ProNTP303, pAGT707, Oligo NLS_F+R, pICH41308-roGFP2-Orp1-SKL, pICH41421	*1*	*ProNTP303:NLS-roGFP2-Orp1-NOS*	*Arnaud et al., 2023*
pICH47811, pICH87644, pICH41308-Hygro, pICH44300	*1*	*Act2:Hygro-TerAct2*	*Arnaud et al., 2023*
pAGM4723, pICH41744, ProNTP303:roGFP2-Orp1-Nos, Act2:Hygro-TerAct2	*2*	*ProNTP303:roGFP2-Orp1-NOS-Act2:Hygro-TerAct2*	*Arnaud et al., 2023*
pAGM4723, pICH41744, ProNTP303:MTS-roGFP2-Orp1-Nos, Act2:Hygro-TerAct2	*2*	*ProNTP303:MTS-roGFP2-Orp1-NOS-Act2:Hygro-TerAct2*	*Arnaud et al., 2023*
pAGM4723, pICH41744, ProNTP303:Chl-roGFP2-Orp1-Nos, Act2:Hygro-TerAct2	*2*	*ProNTP303:Chl-roGFP2-Orp1-NOS-Act2:Hygro-TerAct2*	*Arnaud et al., 2023*
pAGM4723, pICH41744, ProNTP303:roGFP2-Orp1-SKL-Nos, Act2:Hygro-TerAct2	*2*	*ProNTP303:roGFP2-Orp1-SKL-NOS-Act2:Hygro-TerAct2*	*Arnaud et al., 2023*
pAGM4723, pICH41744, NLS-ProNTP303:roGFP2-Orp1-Nos, Act2:Hygro-TerAct2	*2*	*ProNTP303:NLS-roGFP2-Orp1-NOS-Act2:Hygro-TerAct2*	*Arnaud et al., 2023*

Table S3. Multiple reaction monitoring (MRM) transitions for LC-triple quadrupole MS/MS analysis of TCA cycle acids and glutathione.

Compound Name	Quantifier or qualifier ion	Precursor Ion	MS1 Res	Product Ion	MS2 Res	Dwell	Fragmentor (V)	Collision Energy (V)	Cell Accelerator Voltage	Polarity
Succinate	Quantifier	329	Unit	91	Unit	200	135	20	4	Positive
Succinate	Qualifier	329	Unit	206	Unit	200	135	20	4	Positive
Malate	Quantifier	345	Unit	91	Unit	200	135	20	4	Positive
Malate	Qualifier	345	Unit	222	Unit	200	135	20	4	Positive
2-Oxoglutarate	Quantifier	462	Unit	91	Unit	200	135	20	4	Positive
2-Oxoglutarate	Qualifier	462	Unit	339	Unit	200	135	20	4	Positive
Aconitate	Quantifier	490	Unit	91	Unit	200	135	20	4	Positive
Aconitate	Qualifier	490	Unit	325	Unit	200	135	20	4	Positive
Citrate_Isocitrate	Quantifier	508	Unit	91	Unit	200	135	20	4	Positive
Citrate_Isocitrate	Qualifier	508	Unit	385	Unit	200	135	20	4	Positive
Glutathione (GSH)	Quantifier	413	Unit	91	Unit	200	135	20	4	Positive

References

Arnaud D, Deeks MJ, Smirnoff, N. Organelle-targeted biosensors reveal distinct oxidative events during pattern-triggered immune responses. *Plant Physiology*. 2023:191(4):2551–2569. https://doi.org/10.1093/plphys/kiac603

Carrie C, Venne AS, Zahedi, RP, Soll J. Identification of cleavage sites and substrate proteins for two mitochondrial intermediate peptidases in *Arabidopsis thaliana*. *J Exp Bot*. 2015:66(9):2691-2708. https://doi.org/10.1093/jxb/erv064

de Graaf BH, Vatovec S, Juarez-Diaz JA, Chai L, Kooblall K, Wilkins KA., Zou H, Forbes T, Franklin FC, Franklin-Tong VE. The *Papaver* self-incompatibility pollen *S*-determinant, PrpS, functions in *Arabidopsis thaliana*. *Curr Biol*. 2012:22(2):154-159. https://doi.org/10.1016/j.cub.2011.12.006

Engler C, Youles M, Gruetzner R, Ehnert TM, Werner S, Jones JD, Patron NJ, Marillonnet S. A golden gate modular cloning toolbox for plants. *ACS Synth Biol.* 2014:3(11):839-843. https://doi.org/10.1021/sb4001504

Kalderon D, Richardson WD, Markham AF, Smith AE. Sequence requirements for nuclear location of simian virus 40 large-T antigen. *Nature.* 1984:311(5981):33-38. https://doi.org/10.1038/311033a0

Marillonnet S, Giritch A, Gils M, Kandzia R, Klimyuk V, Gleba Y. In planta engineering of viral RNA replicons: efficient assembly by recombination of DNA modules delivered by *Agrobacterium*. *Proc Natl Acad Sci U S A*. 2004:101(18):6852-6857. https://doi.org/10.1073/pnas.0400149101

Sjöling S, Glaser E. Mitochondrial targeting peptides in plants. *Trends in Plant Science.* 1998:3(4):136-140. https://doi.org/10.1016/S1360-1385(98)01212-6

Sparkes IA, Hawes C, Baker A. AtPEX2 and AtPEX10 are targeted to peroxisomes independently of known endoplasmic reticulum trafficking routes. *Plant Physiol. 2005:139*(2):690-700. https://doi.org/10.1104/pp.105.065094

Wen W, Meinkoth JL, Tsien RY, Taylor SS. Identification of a signal for rapid export of proteins from the nucleus. *Cell.* 1995:82(3):463-473. https://doi.org/10.1016/0092-8674(95)90435-2
